# Cost-utility analysis of community occupational therapy in dementia (COTiD-UK) versus usual care: Results from VALID, a multi-site randomised controlled trial in the UK

**DOI:** 10.1371/journal.pone.0262828

**Published:** 2022-02-11

**Authors:** Elena Pizzo, Jennifer Wenborn, Jane Burgess, Jacqueline Mundy, Martin Orrell, Michael King, Rumana Omar, Stephen Morris

**Affiliations:** 1 Department of Applied Health Research, University College London, London, United Kingdom; 2 Division of Psychiatry, University College London, London, United Kingdom; 3 Research & Development Department, North East London NHS Foundation Trust (NELFT), London, United Kingdom; 4 Essex Stroke Hub Team, North East London NHS Foundation Trust (NELFT), London, United Kingdom; 5 Institute of Mental Health, University of Nottingham, Nottingham, United Kingdom; 6 Priment Clinical Trials Unit, Faculty of Brain Sciences, University College London, London, United Kingdom; 7 Department of Statistical Science, University College London, London, United Kingdom; 8 Department of Public Health and Primary Care, University of Cambridge, Cambridge, United Kingdom; Murdoch University, AUSTRALIA

## Abstract

**Background:**

A community-based occupational therapy intervention for people with mild to moderate dementia and their family carers: the Community Occupational Therapy in Dementia–UK version (COTiD-UK); and Treatment as usual (TAU) were randomly assigned to 468 pairs (each comprising a person with dementia and a family carer) in the Valuing Active Life in Dementia (VALID) randomised controlled trial (RCT).

**Objectives:**

To compare the cost-utility of the COTiD-UK intervention compared to TAU, using data from the VALID RCT.

**Methods:**

We performed a cost-utility analysis estimating mean costs and quality adjusted life years (QALYs) per person with dementia and carer for both treatments over a 26 weeks’ time horizon based on resource use data and utility values collected in the trial.

**Results:**

Taking the National Health Service and Personal Social Services perspective, including costs and benefits to the person with dementia only, measuring Health Related Quality of Life based on Dementia Quality of Life scale (DEMQOL), accounting for missing data and adjusting for baseline values, there was a significant difference in costs between COTiD-UK and TAU (mean incremental cost for COTiD-UK £784 (95% CI £233 to £1334)), but no significant difference in outcomes (mean QALYs gained 0.00664 (95% CI -0.00404, 0.01732)). The Incremental Net Monetary Benefit (INMB) for COTiD-UK versus TAU was negative at a maximum willingness to pay for a QALY of £20000 (mean -£651, 95% CI -£878 to -£424) or £30000 (mean -£585, 95% CI -£824 to -£345). Extensive sensitivity analyses confirmed the results.

**Conclusions:**

This community-based occupational therapy intervention has a very low probability of being cost-effective.

## Introduction

In the UK around 850,000 people live with dementia [1], mostly in the community. By 2040, there will be over 1.2million people living with dementia in England and Wales [2], largely due to increased life expectancy and an increased proportion of older people in the population. The current cost of dementia to the UK NHS, local authorities and families is estimated at £26 billion per year, but this is estimated to grow [3]. Approximately 670,000 family members and friends provide support and care to people with dementia, saving up to £11 billion per year [4]. The guidelines for supporting people with dementia and their carers [5] recommend advice and skills training from an occupational therapist to help maintain independence.

A community-based occupational therapy intervention for people with mild to moderate dementia and their family carers–the Community Occupational Therapy in Dementia (COTiD)—was found to be clinically and cost effective in the Netherlands [6–8], but not in Germany [9]. The UK version of the intervention, COTiD-UK has been recently evaluated in the Valuing Active Life in Dementia (VALID) randomised controlled trial (RCT) [10].

The main aim of this study was to assess the costs, outcomes and the cost-utility of the COTiD-UK intervention compared to Treatment as usual (TAU), using data from the VALID RCT.

## Materials and methods

### VALID trial

VALID was a UK multi-centre, parallel-group, pragmatic randomised trial to estimate the clinical and cost-effectiveness of COTiD-UK relative to treatment as usual (TAU) [10]. Trial Registration: Current Controlled Trials ISRCTN10748953. NHS wide ethical approval was obtained from the NRES Committee London–Camberwell St Giles (reference number 14/LO/0736) in June 2014. The CONSORT flow diagram is available in S1 Fig.

Pairs of people (a person with mild to moderate dementia plus an identified family carer) were recruited across 14 sites between September 2014 and July 2017. The former had to: live in their own home; have a diagnosis of dementia as defined by the DSM-IV [11]; and score between 0.5 and 2 on the Clinical Dementia Rating Scale indicating mild to moderate dementia [12]. Carers had to: be aged 18 or over; and provide practical support with domestic or personal activities to the person with dementia for at least four hours per week. Both parties had to: be able to converse in English; be willing to participate in the COTiD-UK intervention together if allocated to receive it; and have the capacity to provide consent.

Pairs were, randomly assigned to either the COTiD-UK or the TAU.

### Interventions

#### Community Occupational Therapy in Dementia (COTiD-UK)

COTiD-UK consists of up to ten hours of community occupational therapy delivered to the pair together over ten weeks. Initially, the occupational therapist conducts a one-to-one narrative interview with each person; assesses the home environment; and observes the person with dementia completing a familiar activity. Next, the occupational therapist summarises the information collected and facilitates a discussion with the pair to identify, agree and prioritise individual and joint lifestyle goals. The therapist then supports the pair to enact their goals, and coaches the carer to develop problem-solving skills and coping strategies. The sessions usually take place where the person with dementia lives but depending on the activities chosen, may also happen in the local community, for example the sports club or garden centre. At the final session the pair and therapist evaluate their success in achieving their goals, and make future plans accordingly.

#### Treatment as Usual (TAU)

Treatment as usual comprises the usual service provided locally within the research site, which could include standard occupational therapy. Services available to people with dementia and their carers varied between and within sites, so each site completed a template detailing usual treatment. To reduce contamination between the two groups, the occupational therapists trained to deliver COTiD-UK were asked not to provide occupational therapy to those pairs allocated to TAU; nor to share their COTiD-UK training or materials with occupational therapists not so trained. At each site an unmasked researcher monitored contamination by checking whether TAU pairs reported contact with COTiD-UK trained occupational therapists.

Outcome data were collected at baseline, then 12, 26, 52 and 78 weeks post randomisation. The primary outcome was the Bristol Activities of Daily Living Scale (BADLS) [13] measured at 26 weeks. Secondary outcomes for the person with dementia included: the Mini Mental State Examination to measure cognition, the EuroQoL (EQ-5D-5L) questionnaire to measure health-related quality of life, the Dementia Quality of Life (DEMQOL) scale, and the DEMQOL-Proxy (completed by the carer), the Interview of Deterioration in Daily activities in Dementia (IDDD) and the Cornell Scale for Depression in Dementia (CSDD). Secondary outcomes for carers included: the EQ-5D-5L, the Sense of Competence Questionnaire (SCQ), the Hospital Anxiety and Depression Scale (HADS).

The evaluation of the clinical effectiveness demonstrated that providing community occupational therapy as provided within the VALID trial did not improve daily activity performance. It also did not improve the secondary outcomes, cognition, mood or quality of life in people with dementia [14].

### Overview of economic evaluation

While previous work [14] has focused specifically on the clinical impact of COTiD-UK, in this work we undertook a cost-utility analysis to compare both the costs and outcomes associated with COTiD-UK versus Treatment as Usual (TAU). This analysis was preferred to a cost-benefit analysis because in general it is very difficult and not appropriate to measure the benefits of a health care intervention in monetary terms. Indeed, the outcome measure used here was quality-adjusted life years (QALYs), which combine length of life and quality of life, and is consistent with the National Institute for Health and Care Excellence (NICE) recommendations [15]. Cost-effectiveness was expressed as incremental net monetary benefits. The base case analysis took a UK National Health Service (NHS) and personal social services (PSS) perspective, though in additional analyses a societal perspective was also undertaken [15]. Source use data were included from all participating centres and UK unit costs were applied. Costs were calculated in 2016/2017 pound sterling (GBP) and inflated where appropriate [16]. The time horizon was 26 weeks, reflecting the main outcomes follow-up in the trial, and was the longest time period over which data were collected for all participants. Extrapolation beyond the end of the trial was not undertaken because the within-trial analysis found a significant incremental cost for COTiD-UK compared to TAU, but no significant difference in outcomes between the two groups; 26 weeks was long enough to reflect all important differences in costs or outcomes between the two interventions. Given the time horizon, discounting was not applied to costs or outcomes. In the base case analysis we investigated cost-utility using the costs and outcomes for the person with dementia (pwd) only; in additional analyses we also included costs and outcomes of the carer.

### Resource use and costs

We assessed the cost of the COTiD-UK set up and training occupational therapists (OTs) in each setting, including venue hiring, refreshment, trainers cost (time, transport and accommodation), occupational therapists costs (time, paid expenses and materials (audio recorder, memory sticks and cost to deliver by post, bag, OPHI, audio feedback and supervision) (S1 Appendix and S1 Table). The cost of two trainers included the cost of their time, travel costs and overnight subsistence.

A total of 44 occupational therapists attended the training, of whom 32 proceeded to the trial and were allocated at least one pair each, although one was subsequently unavailable to provide the intervention as planned due to ill health. We assessed the cost of occupational therapists’ time by multiplying their specific salary per hour [17] by the number of hours attended. These costs are not included in the cost-utility analysis because they are a sunk cost: being incurred as part of the set-up of the programme they cannot be recovered and therefore should not be included in deciding whether to continue the implementation of the intervention. Also, data are not available (on relevant patients seen during remaining years of working life) to estimate accurately the mean cost per patient.

For every pair (person with dementia and carer) we assessed the cost of the COTiD-UK intervention, using trial data on number of sessions, occupational therapists’ time, transport time and cost (S2 Appendix and S2 Table). The total cost of the sessions was assessed by multiplying the total time by the cost per hour of each occupational therapist [17]. The cost of occupational therapists’ transport was assessed using the cost provided by the occupational therapist when using public transport, or by multiplying the miles (return trip) by the cost per mile when using the car. We used the NHS reimbursement unit cost data [18].

For every person with dementia and their carer we estimated the NHS and PSS service use cost (including GP, practice and community nurse, inpatient, outpatient and day case visits for specialist care, occupational therapy and physiotherapy, social worker, nursing home, domestic home help, meals on wheels and day care); medications; adaptations, equipment and continence products required as a result of dementia; and, changes in accommodation (e.g. residential care) (S3 Appendix). For each person with dementia and their carer, the cost of health care resource use was assessed multiplying the number of contacts by the unit cost of each contact. For each person the cost of medications was assessed, multiplying the reported dosage by the unit cost of each medication using the data in the British National Formulary [19]. The cost of changes in accommodation were assessed using the number of days spent by each person with dementia by the cost per day in each setting. The cost of adaptations, equipment and continence products were assessed using the data reported in the CSRI and the most accurate unit cost source obtained from market sources [20].

Where a societal perspective was adopted we included additional costs for the person with dementia (productivity losses due to illness and out of pocket payments for health care service, adaptations and equipment) and carers (productivity losses due to informal care time, transport costs and out of pocket payments for health care services). Productivity losses were assessed by multiplying the days off work by the average cost of one day of work (salary) [21]. Transport costs were assessed using the data from the CSRI about the transport used and the reported costs or the miles per unit cost per mile.

Resource use data were collected retrospectively using an adapted version of the CSRI at baseline (covering the previous 12 weeks), 12 weeks and 26 weeks post-randomisation. Unit costs were taken from published sources and reported (S3 Appendix and S3 Table) [16–23].

### Utilities and quality adjusted life years

Generic health related quality of life was measured at baseline (randomisation), 12 weeks and 26 weeks post-randomisation using the following questionnaires: EQ-5D-5L (both for the person with dementia and carer) [24, 25]; the DEMQOL [26] and DEMQOL-Proxy [27] for the person with dementia only (the DEMQOL-Proxy was completed by the carer). Each EQ-5D-5L health state was converted into a single summary index (utility value) applying a formula that attaches weights to each of the levels in each dimension based on valuations by general population samples [28]. The same method was applied using the DEMQOL and DEMQOL-Proxy profiles for the person with dementia [29]. For the EQ-5D-5L we used a value set for the UK population to calculate utility values at each time point for every participant [30]. Utility values usually take a value between 0 (death) and 1 (full health). Negative values are also possible if health status is worse than death. We constructed a utility profile for every participant assuming a straight line relation between their utility values at each measurement point. If the person died the utility was recorded zero at time of death. Quality Adjusted Life Years (QALYs) for every participant from baseline to 26 weeks were calculated as the area under the utility profile. In the base case analysis (including the person with dementia only) QALYs are reported using the self-reported DEMQOL.

### Managing missing data

Multiple imputation by chained equations was used to jointly impute missing data for: NHS costs and private costs for both the person with dementia and their carer; utility values at every time point; and, total QALYs. Age, gender, study centre and treatment allocation were included in the imputation models as additional explanatory variables. We combined the estimates across the imputed dataset using Rubin’s rules [31]. We used multivariate normal regression to impute missing values, and generated 20 imputed datasets (as less than 20% of data were missing). In S3 Appendix we report a description of the assumptions we made for medications when names were misspelled or dosage was missing.

### Statistical methods

Mean costs, outcomes and net monetary benefits (NMBs) were compared between all people with dementia in the COTiD-UK and TAU groups. We calculated differences in mean costs and QALYs and incremental NMBs between groups. NMBs for COTiD-UK and TAU were calculated as the mean QALYs per person with dementia multiplied by the maximum willingness to pay for a QALY minus the mean cost per person. Incremental NMBs were calculated as the difference in mean QALYs per person for COTiD-UK versus TAU multiplied by the maximum willingness to pay for a QALY minus the difference in mean cost per person. We used the cost-effectiveness threshold range recommended by NICE (£20000 to £30000 per QALY gained) as the lower and upper limits of the maximum willingness to pay for a QALY [15]. If the incremental NMB is positive (negative) then COTiD-UK (TAU) was preferred on cost-effectiveness grounds. QALYs gained were adjusted for study site, therapist teams and baseline utility values using regression analysis; incremental costs were adjusted for baseline costs and study centre. For each of the 20 imputed datasets we ran 1000 bootstrap replications and combined the results using equations described by Briggs et al. [32] to calculate standard errors around mean values accounting for uncertainty in imputed values, the skewed nature of the cost data and utility values and sampling variation. Standard errors were used to calculate 95% CIs around point estimates. We ran additional analyses where we combined cost and QALY data for people with dementia and their carers; to do this we summed the values for both participants.

### Sensitivity analysis

Cost-effectiveness acceptability curves [33] showing the probability that COTiD-UK was cost-effective compared with TAU at a range of values for the maximum willingness to pay for a QALY were generated based on the proportion of the bootstrap replications across all 20 imputed datasets with positive incremental NMBs [34]. The probability that COTiD-UK was cost-effective at a maximum willingness to pay for a QALY of £20000 and £30000 was reported, based on the proportion of bootstrap replications with positive incremental NMBs at these values. We undertook further sensitivity analyses to evaluate the impact of uncertainty: no adjustment for potential confounders; complete case analysis without imputing missing values with and without adjustment. We performed the analyses adopting NHS/PSS perspectives, using different HRQL measures for the person with dementia, and combining costs and QALYs for the person with dementia with those of their carer (S4 Appendix).

## Results

See supplementary material for a summary of resource use and unit cost data used in the analysis.

The cost of COTiD-UK training for the occupational therapists has been estimated at £96469 (£3000 per therapist), including venue hiring, trainers’ costs, occupational therapists’ and supervisors’ attendance, additional material and audio feedback supervision (S1 Appendix and S1 Table).

The mean cost of the COTiD-UK intervention per pair was estimated to be £619, including the sessions’ time and transport cost (S2 Appendix and S2 Table).

Taking an NHS and PSS perspective, including costs and benefits to the person with dementia only, including the mean cost of the COTiD-UK intervention per pair, measuring HRQL based on DEMQOL and accounting for missing data, the mean total NHS and PSS cost per person with dementia (95% CI) were £2689 (£2272 to £3105) in the COTiD-UK group and £1919 (£1488 to £2349) in the TAU group (Table 1). Mean total QALYs per person with dementia (95% CI) were 0.320 (0.311 to 0.328) in the COTiD-UK group and 0.310 (0.302 to 0.317) in the TAU group (Table 1).

**Table 1:** Mean utility values, QALYs and costs per person with dementia.

	COTiD-UK		Treatment as usual	
	Mean	(95% CI)	Mean	(95% CI)
Total NHS&PSS cost pwd	2689	(2272, 3105)	1919	(1488, 2349)
Total NHS&PSS cost both	2735	(2317, 3153)	1963	(1532, 2394)
Total Societal cost pwd	3983	(3402, 4565)	2922	(2364, 3479)
Total Societal cost both	4121	(3527, 4714)	3130	(2547, 3713)
QALYs (EQ-5D) pwd	0.389	(0.376, 0.403)	0.372	(0.357, 0.388)
QALYs (EQ-5D) both	0.786	(0.767, 0.805)	0.778	(0.749, 0.788)
QALYs (DEMQOL) pwd	0.320	(0.311, 0.328)	0.310	(0.302, 0.317)
QALYs (DEMQOL, EQ-5D) both	0.721	(0.705, 0.736)	0.704	(0.690, 0.718)
QALYs (DEMQOL-Proxy) pwd	0.333	(0.325, 0.341)	0.331	(0.321, 0.341)
QALYs (DEMQOL-Proxy, EQ-5D) both	0.729	(0.714, 0.743)	0.730	(0.715, 0.745)

pwd: person with dementia; both: include both person with dementia and carers

QALY: Quality Adjusted Life Year; CI: confidence interval

Notes: For person with dementia QALYs are calculated using the EQ-5D-5L, the DEMQOL and the DEMQOL-Proxy

for carers QALYs are calculated using the EQ-5D-5L questionnaire only; QALYs in the “both” scenarios are calculated as the sum of the QALYs for person with dementia and carers. Costs are in 2016/2017 Pounds sterling (GBP). Data include values imputed using multiple imputation (see text). The 95% confidence intervals were derived from 1000 bootstrap replications of each of the 20 imputed datasets (see text).

**Fig 1:**
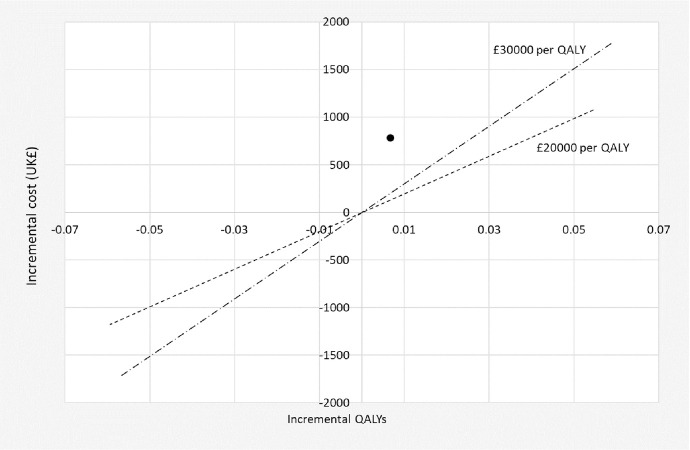
Cost-effectiveness plane and Incremental Cost-Effectiveness Ratio of COTiD-UK vs TAU. Cost-effectiveness plane showing that the Incremental Cost-Effectiveness Ratio (ICER) of COTiD-UK vs TAU is always above the lower (£20,000 per QALY) and upper (£30,000 per QALY) willingness to pay threshold for a QALY. Base case results including NHS costs for the person with dementia, QALYs measured using DEMQOL (black circle).

In the base case analysis, using the above results and adjusting for baseline values, there was a significant difference in costs between the two groups (mean incremental cost for COTiD-UK £784 (95% CI £233 to £1334; p-value 0.005)), but no significant difference in outcomes (mean QALYs gained 0.00664 (95% CI -0.00404, 0.01732; p-value 0.222)) (Table 2). The difference in cost and the difference in outcomes (QALYs) of the COTiD-UK versus TAU is represented in the Cost-effectiveness Plane (Fig 1): it is evident that the Incremental Cost-Effectiveness Ratio (ICER) of the intervention is above the two thresholds of £20000 and £30000 per QALY (the two dotted lines) recommended by NICE in the UK for the intervention to be acceptable (results for other perspectives are in Appendix, S3a–S3d Fig). The Incremental Net Monetary Benefit (INMB) for COTiD-UK versus TAU was negative at a maximum willingness to pay for a QALY of £20000 (mean -£651, 95% CI -£878 to -£424) or £30000 (mean -£585, 95% CI -£824 to -£345).

**Table 2:** Incremental cost-effectiveness of COTiD-UK vs. TAU.

	Incremental cost			QALYs gained	Incremental Net Monetary Benefit			
					£20,000		£30,000	
NHS&PSS cost, DEMQOL, pwd	Mean	95% CI	Mean	95% CI	Mean	95% CI	Mean	95% CI
Base case ^a^	784	(233, 1334)	0.00664	(-0.00404, 0.01732)	-651	(-878, -424)	-585	(-824, -345)
No adjustment ^b^	769	(171, 1368)	0.00965	(-0.001802, 0.02110)	-576	(-823, -329)	-480	(-740,-219)
Complete case analysis ^c^	838	(274, 1402)	0.00731	(-0.003363, 0.01798)	-692	(-911, -472)	-619	(-854, -384)
Complete case analysis, no adjustment ^d^	1047	(441, 1653)	0.01060	(-0.000773, 0.02196)	-835	(-1071, -599)	-729	(-981, -477)
**Other perspectives:**								
NHS&PSS cost, EQ-5D, pwd (Base case^a^)	784	(233, 1334)	0.01298	(-0.00089, 0.0268)	-524	(-745, -303)	-394	(-642, -147)
NHS&PSS cost, DEMQOL-Proxy, pwd (Base case^a^)	784	(233, 1334)	0.00063	(-0.00994, 0.0112)	-771	(-996, -547)	-765	(-1002, -528)
Societal cost, EQ-5D, pwd (Base case^a^)	951	(253, 1650)	0.01298	(-0.00089, 0.0268)	-692	(-962, -422)	-562	(-854, -270)
Societal cost, DEMQOL, pwd (Base case^a^)	951	(253, 1650)	0.00664	(-0.00404, 0.01732)	-818	(-1102, -535)	-752	(-1046, -458)
Societal cost, DEMQOL-Proxy, pwd (Base case^a^)	951	(253, 1650)	0.00063	(-0.00994, 0.0112)	-939	(-1219, -659)	-933	(-1223, -642)
NHS&PSS cost, EQ-5D, both (Base case^a^)	793	(241, 1344)	0.01202	(-0.00452, 0.02856)	-552	(-783, -322)	-432	(-698, -166)
NHS&PSS cost, DEMQOL, both (Base case^a^)	793	(241, 1344)	0.01091	(-0.0035, 0.025325)	-574	(-810, -339)	-465	(-723, -208)
NHS&PSS cost, DEMQOL-Proxy, both (Base case^a^)	793	(241, 1344)	-0.00355	(-0.01792, 0.01082)	-864	(-1097, -630)	-899	(-1155, -643)
Societal cost, EQ-5D, both (Base case ^a^)	853	(156, 1550)	0.01202	(-0.00452, 0.02856)	-613	(-890, -336)	-493	(-800, -185)
Societal cost, DEMQOL, both (Base case ^a^)	853	(156, 1550)	0.01091	(-0.0035, 0.025325)	-635	(-925, -345)	-526	(-833, -218)
Societal cost, DEMQOL-Proxy, both (Base case ^a^)	853	(156, 1550)	-0.00355	(-0.01792, 0.01082)	-924	(-1204, -645)	-960	(-1250,-670)

(a) Data include values imputed using multiple imputation (see text). The QALYs gained, incremental cost and incremental NMB figures are for COTiD-UK minus TAU and are adjusted for potential confounders (see text).

(b) As for the base case analysis except the QALYs gained and costs are unadjusted.

(c) As for the base case analysis except there is no multiple imputation of missing values.

(d) As for c but the analysis is unadjusted.

**Fig 2:**
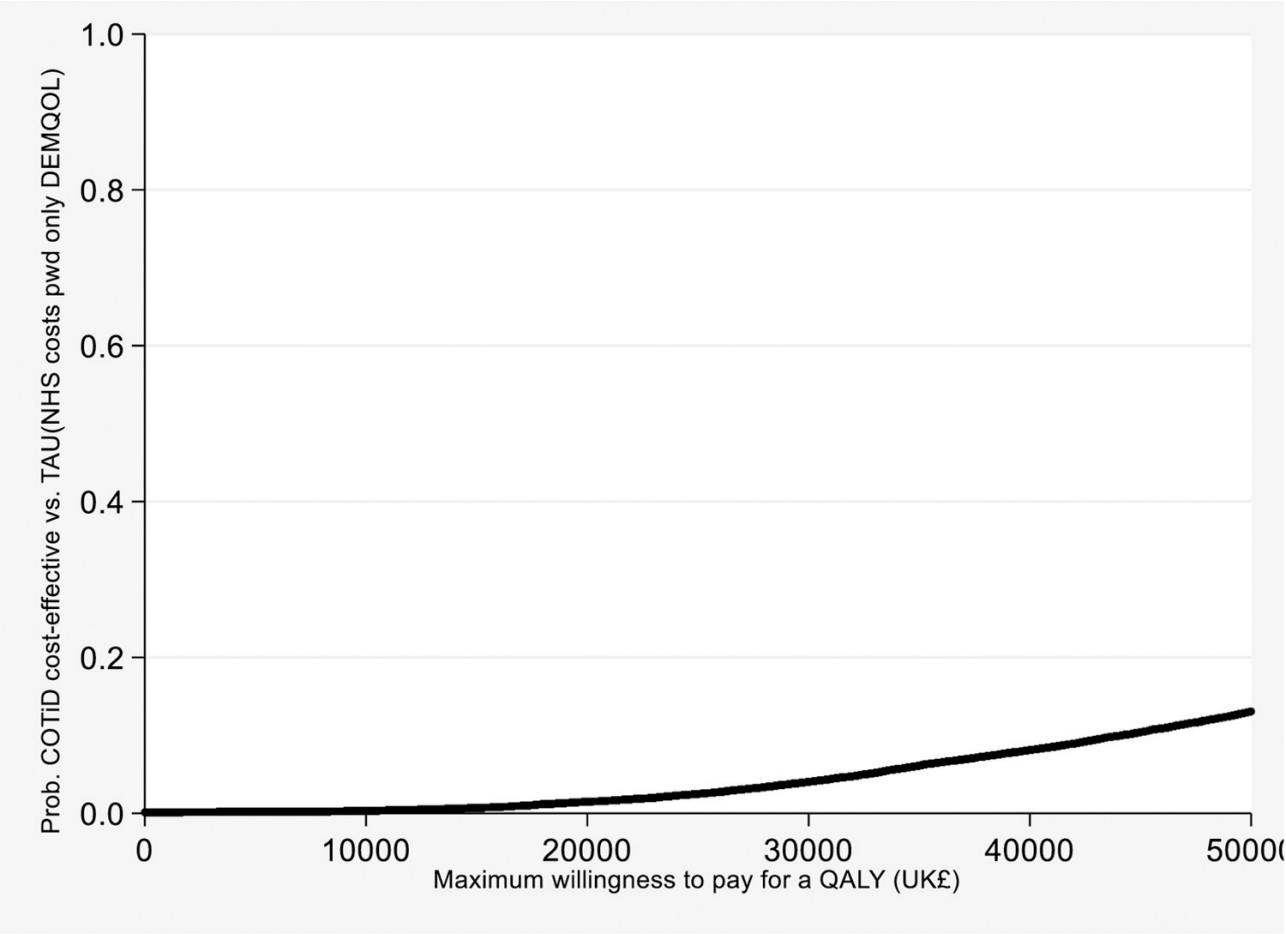
Cost-effectiveness acceptability curve showing the probability that COTiD-UK vs TAU is cost-effective. Cost-effectiveness acceptability curve showing the probability that COTiD-UK vs TAU is cost-effective at different values of the maximum willingness to pay for a QALY. The probability TAU is cost-effective is one minus the probability COTiD-UK is cost-effective at each value of the maximum willingness to pay for a QALY. Base case results including NHS costs for the person with dementia, QALYs measured using DEMQOL.

At the maximum willingness to pay for a QALY of £20 000 (£30 000) the probability that the COTiD-UK was cost-effective was 2% (4%) (Fig 2). The results were not significantly different when undertaking the analysis without adjustment, and using complete cases (Table 2).

The findings were qualitatively the same (significantly higher costs associated with COTiD-UK, no significant differences in QALYs) when adopting NHS/PSS and societal perspective, using different HRQL measures for the person with dementia to estimate QALYs (EQ-5D-5L, DEMQOL-Proxy) and combining costs and QALYs for the person with dementia with those of their carer (S4 Appendix, S4–S14 Tables and S2 Fig).

We did run several statistical regressions to control for age, gender, marital status and sites of the intervention to see if the intervention could be cost-effective in a sub-group of the population but the results were not statistical significant.

## Discussion

Our economic analysis of the VALID trial data showed that COTiD-UK was more expensive than treatment as usual and didn’t significantly improve health outcomes in people with dementia or their family carers over six months follow-up. Comprehensive sensitivity analyses showed little uncertainty in these findings. The findings mean that the COTiD-UK intervention is not cost-effective at the maximum willingness to pay for a QALY of £20000 or £30000.

A previous economic evaluation of COTiD has shown that it was cost-effective in the Netherlands [8]. This study differs to ours in that the control group did not receive any occupational therapy, whereas in our study the treatment as usual may have included some occupational therapy depending on the usual care provided within each research site (S3 Appendix and S3 Table) and that the time horizon in our study was three months longer: this might have an impact on health outcomes. Similarly to our study, the evaluation of COTiD in Germany has shown that the intervention was not cost-effective [9].

The main strength of our analysis is that it is based on a large multicentre randomised trial with extremely detailed information on resource use, utility values captured using at least three different questionnaires and mortality. We also adopted different study perspectives, used several measures to estimate QALYs and ran analyses for people with dementia and their carers and the results were consistent in every case.

Our study has several limitations. First, resource use data were collected retrospectively (referring to the previous 12 weeks) and this might have caused some recollection problems on resources used; however, there is no reason to believe this problem would differ between study groups. Second, we had to make some assumptions about the unit costs used to assess the NHS resource use. For A&E admissions, hospital admissions, and outpatient visits we used an average cost for each type of contact as most of the reasons for admissions were not clearly stated; this may have affected the results if the reasons for each type of contact varied between COTiD-UK and TAU. However, extensive sensitivity analysis was conducted to take this into account, but differences in unit cost data (e.g. using median costs instead of average costs) won’t change the final result, being the analysis incremental. Due to missing data, several assumptions had to be made regarding the cost of medications, the dosage and unit costs. Third, carers were asked to record only their use of psychotropic medications as mental health can be linked to care activities; we did not include any other medications taken by carers, (e.g. painkillers) though these may be related to caring activities. Fourth, in some analyses we combined (summed) the QALYs for people with dementia and their carers, even though they were measured using different questionnaires (DEMQOL or DEMQOL-Proxy for person with dementia and EQ-5D-5L for carers) and therefore captured different aspects of HRQOL. Fifth, the time horizon was 26 weeks. We could have taken a shorter time horizon to see the immediate effect, or a longer time horizon, but it is unlikely that this would have changed the results given there were no significant differences in costs and outcomes at 26 weeks.

According to the results of our analysis it would require a willingness to pay higher than £120,000 per QALY gained to render the intervention cost-effective. This could be the case in the United States, where the threshold values are more generous, in line with higher spending on medical care [35]. Though, it is speculative to assume this, given that costs and outcomes differ across countries and the results of this analysis are based on the UK values.

In conclusion, our analysis has shown that the probability of COTiD-UK being cost-effective is very low in the UK setting, at the current willingness to pay thresholds.

## Supporting information

S1 Fig CONSORT diagram showing flow of pairs through the trial. (DOCX)

S2 Fig Cost-effectiveness acceptability curve showing the probability that COTiD-UK vs TAU is cost-effective at different values of the maximum willingness to pay for a QALY (a-j). QALY = quality adjusted life year. The probability TUA is cost-effective is one minus the probability COTiD-UK is cost-effective at each value of the maximum willingness to pay for a QALY.
(DOCX)

S3 Fig Cost-effectiveness Plane showing the Incremental Costs and Incremental QALYs of COTiD-UK vs TAU (a-d). QALY = quality adjusted life year. The two dotted lines represent the lower and upper threshold of £20000 and £30000 per QALY. Interventions below the line (threshold) are acceptable, above the line are not acceptable.
(DOCX)

S1 Appendix Cost to train occupational therapists to deliver COTiD-UK. (DOCX)

S2 Appendix Cost of COTiD-UK intervention. (DOCX)

S3 Appendix Summary of data used in the cost-utility analysis. (DOCX)

S4 Appendix Results of cost-utility analysis using different perspectives (NHS or societal) and health care questionnaires (EQ-5D-5L, DEMQOL and DEMQOL-Proxy) for person with dementia. (DOCX)

S1 Table Cost to set-up and train OTs for COTiD-UK. Costs are in 2017 Pounds Sterling (GBP); OT: occupational therapist.
(DOCX)

S2 Table Cost of COTiD-UK intervention (OT time per session and OT transport cost). Costs are in 2017 Pounds Sterling (GBP); OT: occupational therapist.
(DOCX)

S3 Table Part 1–2 resource use, unit costs, utility values, QALYs person with dementia (part 1 & 2). a) average number of contacts/visits per person; b) Duration of each visit/contact in minutes; c) Total number of contacts in the group; Unit costs are in 2017 Pounds sterling (GBP). **Part 3. Resource use, unit costs, utility values, QALYs carer (part 3).** a) average number of contacts/visits per person; b) Duration of each visit/contact in minutes; c) Total number of contacts in the group; Unit costs are in 2017 Pounds sterling (GBP).
(ZIP)

S4 Table Incremental cost-effectiveness of COTiD-UK vs. TAU, NHS costs person with dementia using EQ-5D-5L. a) Data include values imputed using multiple imputation (see text). The QALYs gained, incremental cost and incremental NMB figures are for COTiD-UK minus TAU and are adjusted for potential confounders (see text).; b) As for the base case analysis except the QALYs gained and costs are unadjusted.; c) As for the base case analysis except there is no multiple imputation of missing values.; d) As for c but the analysis is unadjusted.
(DOCX)

S5 Table Incremental cost-effectiveness of COTiD-UK vs. TAU, NHS costs person with dementia using DEMQOL-Proxy. a) Data include values imputed using multiple imputation (see text). The QALYs gained, incremental cost and incremental NMB figures are for COTiD-UK minus TAU and are adjusted for potential confounders (see text).; b) As for the base case analysis except the QALYs gained and costs are unadjusted.; c) As for the base case analysis except there is no multiple imputation of missing values.; d) As for c but the analysis is unadjusted.
(DOCX)

S6 Table Incremental cost-effectiveness of COTiD-UK vs. TAU, societal costs person with dementia using EQ-5D-5L. a) Data include values imputed using multiple imputation (see text). The QALYs gained, incremental cost and incremental NMB figures are for COTiD-UK minus TAU and are adjusted for potential confounders (see text).; b) As for the base case analysis except the QALYs gained and costs are unadjusted.; c) As for the base case analysis except there is no multiple imputation of missing values.; d) As for c but the analysis is unadjusted.
(DOCX)

S7 Table Incremental cost-effectiveness of COTiD-UK vs. TAU, societal costs person with dementia using DEMQOL. a) Data include values imputed using multiple imputation (see text). The QALYs gained, incremental cost and incremental NMB figures are for COTiD-UK minus TAU and are adjusted for potential confounders (see text).; b) As for the base case analysis except the QALYs gained and costs are unadjusted.; c) As for the base case analysis except there is no multiple imputation of missing values.; d) As for c but the analysis is unadjusted.
(DOCX)

S8 Table Incremental cost-effectiveness of COTiD-UK vs. TAU, societal costs person with dementia using DEMQOL-Proxy. a) Data include values imputed using multiple imputation (see text). The QALYs gained, incremental cost and incremental NMB figures are for COTiD-UK minus TAU and are adjusted for potential confounders (see text).; b) As for the base case analysis except the QALYs gained and costs are unadjusted.; c) As for the base case analysis except there is no multiple imputation of missing values.; d) As for c but the analysis is unadjusted.
(DOCX)

S9 Table Incremental cost-effectiveness of COTiD-UK vs. TAU, NHS costs both using EQ-5D-5L. a) Data include values imputed using multiple imputation (see text). The QALYs gained, incremental cost and incremental NMB figures are for COTiD-UK minus TAU and are adjusted for potential confounders (see text).; b) As for the base case analysis except the QALYs gained and costs are unadjusted.; c) As for the base case analysis except there is no multiple imputation of missing values.; d) As for c but the analysis is unadjusted.
(DOCX)

S10 Table Incremental cost-effectiveness of COTiD-UK vs. TAU, NHS costs both using DEMQOL. a) Data include values imputed using multiple imputation (see text). The QALYs gained, incremental cost and incremental NMB figures are for COTiD-UK minus TAU and are adjusted for potential confounders (see text).; b) As for the base case analysis except the QALYs gained and costs are unadjusted.; c) As for the base case analysis except there is no multiple imputation of missing values.; d) As for c but the analysis is unadjusted.
(DOCX)

S11 Table Incremental cost-effectiveness of COTiD-UK vs. TAU, NHS costs both using DEMQOL-Proxy. a) Data include values imputed using multiple imputation (see text). The QALYs gained, incremental cost and incremental NMB figures are for COTiD-UK minus TAU and are adjusted for potential confounders (see text).; b) As for the base case analysis except the QALYs gained and costs are unadjusted.; c) As for the base case analysis except there is no multiple imputation of missing values.; d) As for c but the analysis is unadjusted.
(DOCX)

S12 Table Incremental cost-effectiveness of COTiD-UK vs. TAU, societal costs both using EQ-5D-5L. a) Data include values imputed using multiple imputation (see text). The QALYs gained, incremental cost and incremental NMB figures are for COTiD-UK minus TAU and are adjusted for potential confounders (see text).; b) As for the base case analysis except the QALYs gained and costs are unadjusted.; c) As for the base case analysis except there is no multiple imputation of missing values.; d) As for c but the analysis is unadjusted.
(DOCX)

S13 Table Incremental cost-effectiveness of COTiD-UK vs. TAU, societal costs both using DEMQOL. a) Data include values imputed using multiple imputation (see text). The QALYs gained, incremental cost and incremental NMB figures are for COTiD-UK minus TAU and are adjusted for potential confounders (see text).; b) As for the base case analysis except the QALYs gained and costs are unadjusted.; c) As for the base case analysis except there is no multiple imputation of missing values.; d) As for c but the analysis is unadjusted.
(DOCX)

S14 Table Incremental cost-effectiveness of COTiD-UK vs. TAU, societal costs both using DEMQOL-Proxy. a) Data include values imputed using multiple imputation (see text). The QALYs gained, incremental cost and incremental NMB figures are for COTiD-UK minus TAU and are adjusted for potential confounders (see text).; b) As for the base case analysis except the QALYs gained and costs are unadjusted.; c) As for the base case analysis except there is no multiple imputation of missing values.; d) As for c but the analysis is unadjusted.
(DOCX)

S15 Table Consolidated Health Economic Evaluation Reporting Standards (CHEERS) statement. (DOCX)

## References

[1] Alzheimer’s Society, “Dementia friendly utility guide: a practical guide to supporting your customers and employees affected by dementia,” London, 2018.

[2] Ahmadi-Abhari S, Guzman-Castillo M, Bandosz P, Shipley M, Muniz-Terrera G, Singh-Manoux M, et al., “Temporal trend in dementia incidence since 2002 and projections for prevalence in England and Wales to 2040: modelling study,” BMJ 2017; 358:j2856. doi: 10.1136/bmj.j2856 28679494 PMC5497174

[3] Alzheimer’s Society, “A report into the prevalence and cost of dementia prepared by the Personal Social Services Unit (PSSRU) at the London School of Economics and the Institute of Psychiatry at King’s College London, for the Alzheimer’s society,” London, 2018.

[4] Alzheimer’s Society, “Dementia 2014: Opportunity for change.,” London, 2014.

[5] NICE, “Dementia: assessment, management and support for people living with dementia and their carers.,” www.nice.org.uk/NG97 [last accessed 1 august 2018], 2018. 30011160

[6] Graff M, Vernooij-Dassen M, Thijssen M, Dekker J, Hoefnagels W and Rikkert M, “Community based occupational therapy for patients with dementia and their care givers: randomised controlled trial.,” BMJ 2006; 333:1196–201. doi: 10.1136/bmj.39001.688843.BE 17114212 PMC1693594

[7] Graff M, Vernooij-Dassen M, Thijssen M, Dekker J, Hoefnagels W and Olderikker M, “Effects of community occupational therapy on quality of life and health status in dementia patients and their primary caregivers: a randomized controlled trial.,” J Gerontol A Biol Sci Med Sci 2007; 62(9):1002–9. doi: 10.1093/gerona/62.9.1002 17895439

[8] Graff M, Adang E, Vernooij-Dassen M, Dekker J, Jonsson L, Thijssen M, et al., “Community occupational therapy for older patients with dementia and their care givers: cost-effectiveness study.,” BMJ 2008; 336(7636):134–138. doi: 10.1136/bmj.39408.481898.BE 18171718 PMC2206302

[9] Voigt-Radlof S, Graff M, Leonhart R, Schornstein K, Jessen F, Bohlken J, et al., “A multicentre RCT on community occupational therapy in Alzheimer’s disease: 10 sessions are not better than one consultation.,” BMJ Open 2011; 1(1):e000096. doi: 10.1136/bmjopen-2011-000096 22021760 PMC3191435

[10] Wenborn J, Hynes S, Moniz-Cook E, Mountain G, Poland F, King M, et al., “Community Occupational Therapy for people with dementia and family carers (COTiD-UK) versus treatment as usual (Valuing Active Life in Dementia [VALID] study): study protocol for a randomised controlled trial.,” Trials 2016; 17(65). 10.1186/s13063-015-1150-y PMC4739339 26841799

[11] American Psychiatric Association. Diagnosis and Statistical Manual of mental disorders. (4^th^ edition). Washington DC, USA: American Psychiatric Association; 1994.

[12] Hughes CP, Berg L, Danziger WL, Coben LA, Martin RL. A new clinical scale for the staging of dementia. Br J Psych 1982;140:566–72. doi: 10.1192/bjp.140.6.566 7104545

[13] Bucks R, Ashworth D, Wilcock G and Siegfried K, “Assessment of activities of daily living in dementia: development of the Bristol Activities of Daily Living Scale.,” Age Ageing 1996; 25(2); 113–20. doi: 10.1093/ageing/25.2.113 8670538

[14] Wenborn J, O’Keeffe A, Mountain G, Moniz-Cook E, King M, Omar R, et al., “Community Occupational Therapy for people with dementia and family carers (COTiD-UK) versus treatment as usual (Valuing Active Life in Dementia [VALID]) study: a single-blind, randomised controlled trial.,” PLOS Medicine 2021;18(1): e1003433. doi: 10.1371/journal.pmed.1003433 33395437 PMC7781374

[15] NICE, “Guide to the methods of technology appraisal 2013,” NICE, London, 2013. 27905712

[16] ONS, “ONS series, GDP deflators at market Prices, and money GDP last updated 22 February 2018.,” [Online]. [Accessed April 2018].

[17] Curtis L and Burns A, “Unit Costs of Health & Social Care 2017- PSSRU,” University of Kent, 2017.

[18] NHS, “NHS Employers,” [Online]. Available: https://www.nhsemployers.org/tchandbook/part-3-terms-and-conditions-of-service/section-17-reimbursement-of-travel-costs. [Accessed Aug 2020].

[19] BMA, RPSGB, British National Formulary, Pharmaceutical Press, 2017 March.

[20] AgeUK, “https://www.ageuk.org.uk/northwestkent/our-services/service-price-list/,” [Online]. [Accessed Feb 2018].

[21] ONS, “ONS, Employment and labour market salary,” 2017. [Online]. Available: https://www.ons.gov.uk/employmentandlabourmarket/peopleinwork/earningsandworkinghours. [Accessed Feb 2018].

[22] NHS England, “National Prices and National Tariff Workbook 2016/17,” NHS England, 2017.

[23] Morris S, Patel N, Baio G, Kelly L, Lewis-Holmes E, Omar R, et al., “Monetary costs of agitation in older adults with Alzheimer’s disease in the UK: prospective cohort study 2015; 5:e007382.,” BMJ Open 2015; 5(3):e007382. doi: 10.1136/bmjopen-2014-007382 25770235 PMC4360590

[24] Brooks R, “EuroQol: the current state of play,” Health Policy 1996;37(1):53–72. doi: 10.1016/0168-8510(96)00822-6 10158943

[25] The EuroQol Group, “EuroQol-a new facility for the measurement of health-related quality of life,” Health Policy 1990; 16:199–208. doi: 10.1016/0168-8510(90)90421-9 10109801

[26] Smith S, Lamping D, Banerjee S, Harwood R, Foley B, Smith P, et al., “Measurement of health-related quality of life for people with dementia: development of a new instrument (DEMQOL) and an evaluation of current methodology,” Health Technol Assess 2005; 9(10):1–93. doi: 10.3310/hta9100 15774233

[27] Smith S, Murray J, Banerjee S, Foley B, Cook J, Lamping D, et al., “What constitutes health-related quality of life in dementia? Development of a conceptual framework for people with dementia and their carers,” Int J Geriatr Psychiatry, 2005; 20(9):889–895. doi: 10.1002/gps.1374 16116582

[28] van Hout B, Janssen M, Feng Y, Kohlmann T, Busschbach J, Golicki D, et al., “Interim scoring for the EQ-5D-5L: mapping the EQ-5D-5L to EQ-5D-3L value sets,” Value Health 2012;15(5):708–15. doi: 10.1016/j.jval.2012.02.008 22867780

[29] Rowen D, Mulhern B, Banerjee S, van Hout B, Young T, Knapp M, et al., “Estimating Preference-Based Single Index Measures for Dementia Using DEMQOL and DEMQOL-Proxy,” Value Health 2012;15:346–356. doi: 10.1016/j.jval.2011.10.016 22433767

[30] Devlin N, Shah K, Feng Y, Mulhern B and van Hout B, “Valuing health‐related quality of life: An EQ‐5D‐5L value set,” Health Economics 2017;27(1):1–6. 10.1002/hec.3564 PMC6680214 28833869

[31] Rubin D, Multiple Imputation for Nonresponse in Surveys, New York: Wiley, 1987.

[32] Briggs A, Clark T, Wolstenholme J. and Clarke P., “Missing… presumed at random: cost-analysis of incomplete data,” Health Econ 2003; 12(5):377–392. doi: 10.1002/hec.766 12720255

[33] Briggs A and Gray A, “Handling uncertainty in economic evaluation of healthcare interventions,” BMJ 1999; 319:635. doi: 10.1136/bmj.319.7210.635 10473486 PMC1116497

[34] Stinnett A and J. Mullahy J, “Net health benefits: a new framework for the analysis of uncertainty in cost-effectiveness analysis,” Med Decis Making 1998;18(2 Suppl):S68–S80. 9566468 10.1177/0272989X98018002S09

[35] Thokala P, Carlson J and Drummond M, “HTA’d in the USA: A comparison of ICER in the United States with NICE in England and Wales”, J Manag Care Spec Pharm 2020; 26(9): 1162–1170. doi: 10.18553/jmcp.2020.26.9.1162 32857653 PMC10391099

