# Supplementary figures and images for: Cost-utility analysis of community occupational therapy in dementia (COTiD-UK) versus usual care: Results from VALID, a multi-site randomised controlled trial in the UK
Source: PLoS One. 2022 Feb 11;17(2):e0262828. doi: 10.1371/journal.pone.0262828 (PMC8836304; doi:10.1371/journal.pone.0262828)

**S1 Fig.** **CONSORT diagram showing flow of pairs through the trial**


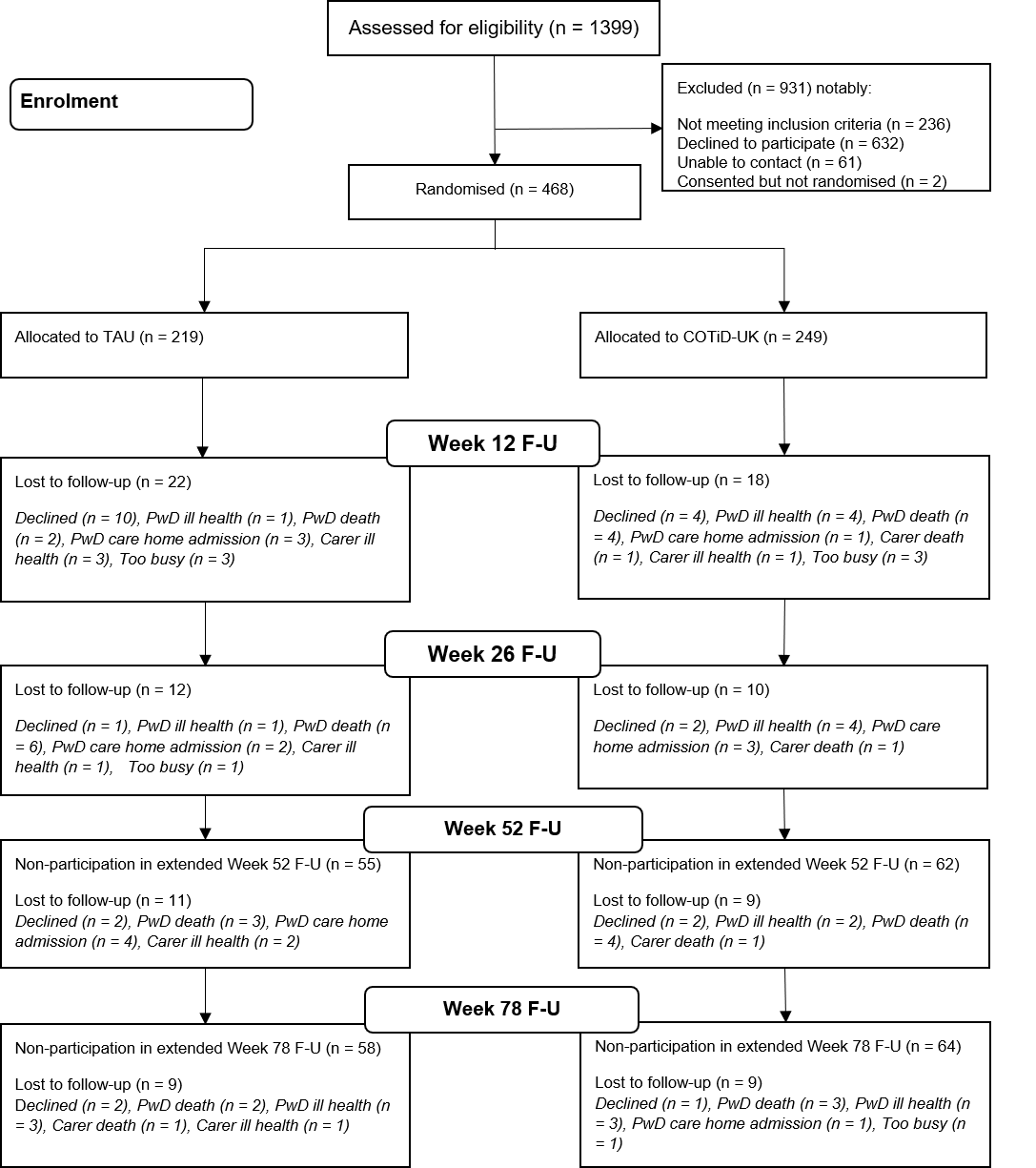

Supplement: S1 Fig — (DOCX) [file pone.0262828.s001.docx]
