# Supplementary material for: Cost-utility analysis of community occupational therapy in dementia (COTiD-UK) versus usual care: Results from VALID, a multi-site randomised controlled trial in the UK
Source: PLoS One. 2022 Feb 11;17(2):e0262828. doi: 10.1371/journal.pone.0262828 (PMC8836304; doi:10.1371/journal.pone.0262828)

**S2 Fig Cost-effectiveness acceptability curve showing the probability that COTiD-UK vs TAU is cost-effective at different values of the maximum willingness to pay for a QALY.**

QALY=quality adjusted life year. The probability TUA is cost-effective is one minus the probability COTiD-UK is cost-effective at each value of the maximum willingness to pay for a QALY.

1. **NHS costs person with dementia only, EQ-5D-5L**


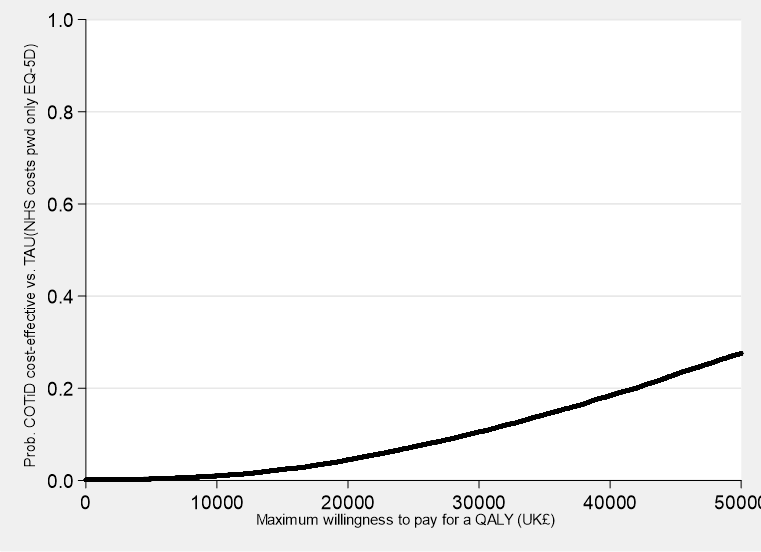


1. **NHS costs person with dementia only, DEMQOL- Proxy**

**c) Societal costs person with dementia only, EQ-5D-5L**


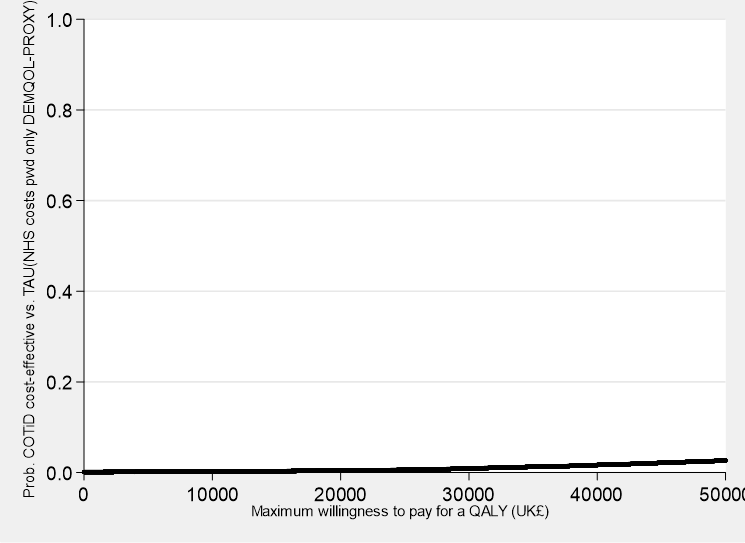

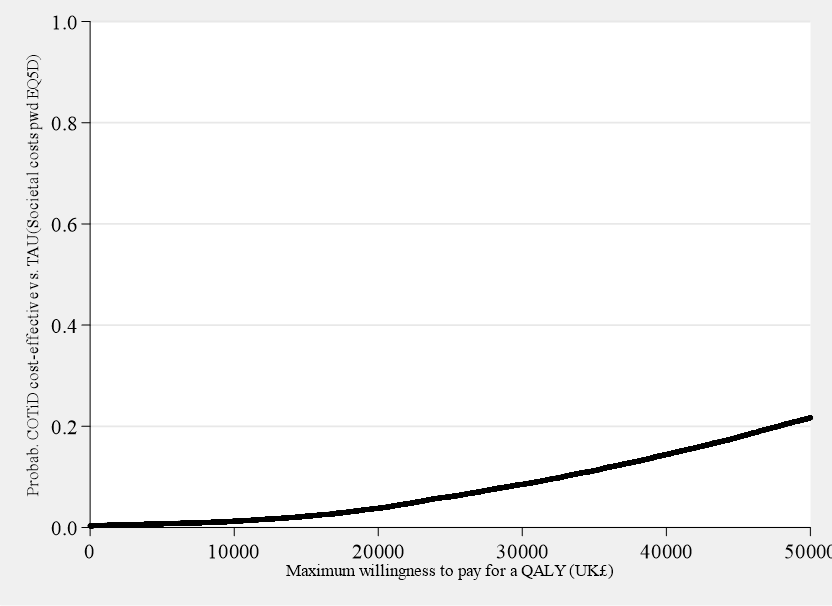


**d) Societal costs person with dementia only, DEMQOL**

**e) Societal costs person with dementia only, DEMQOL-Proxy**


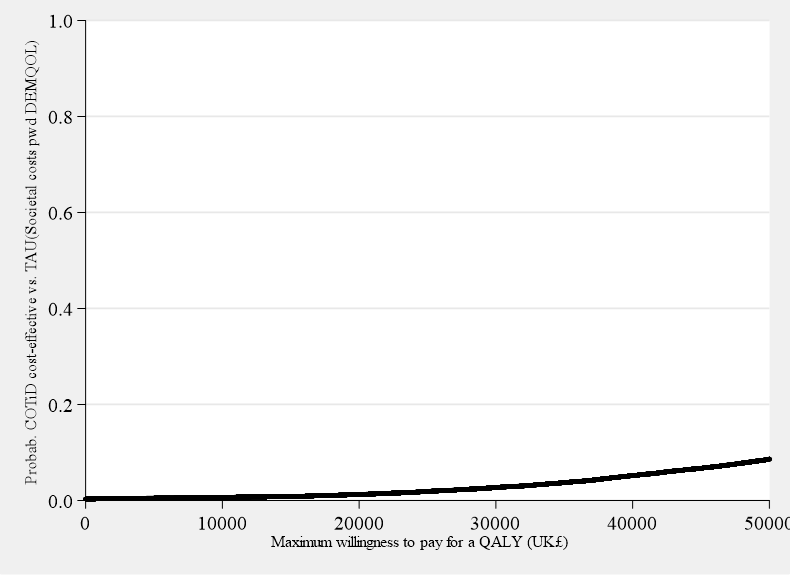

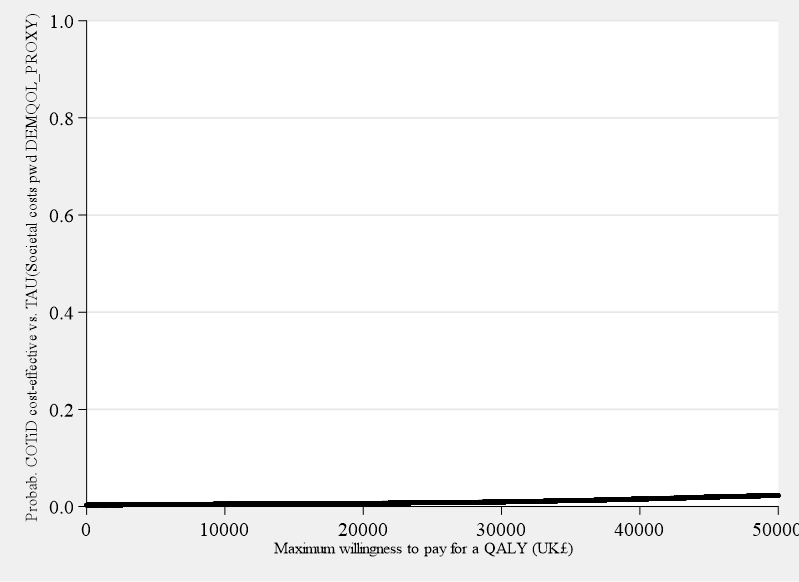


**f) NHS costs both, EQ-5D-5L**


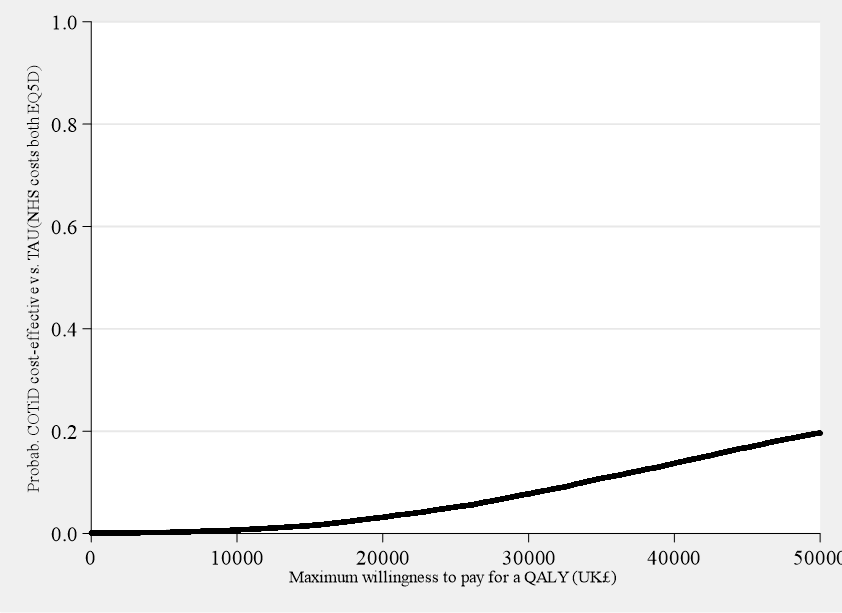


**g) NHS costs both, DEMQOL**


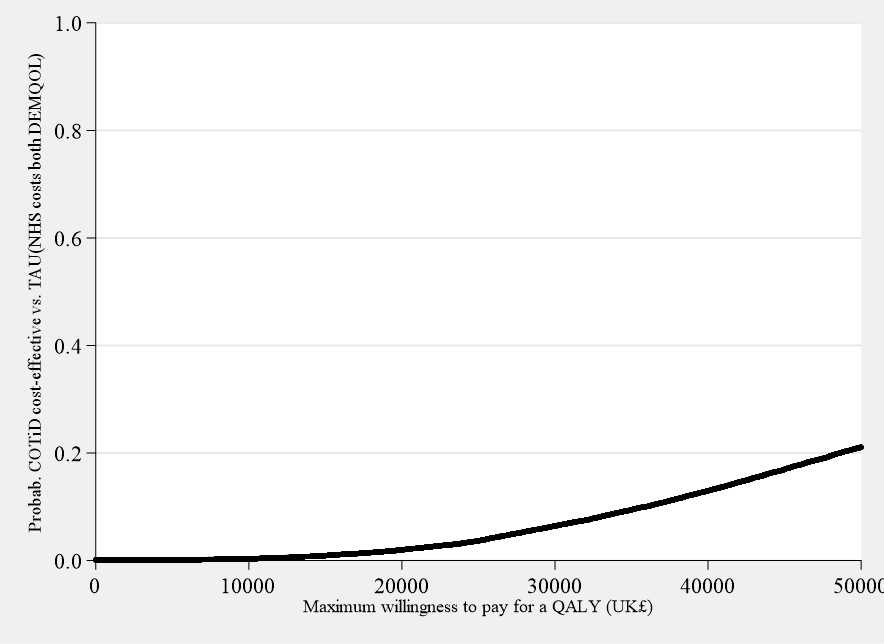


**h) NHS costs both, DEMQOL-Proxy**


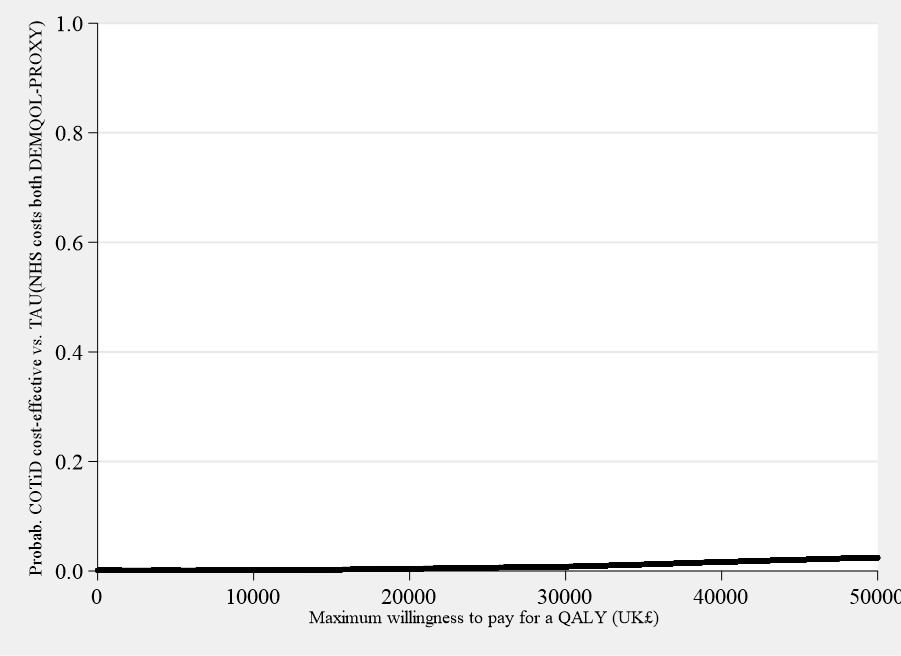


**i) Societal costs both, EQ-5D-5L**


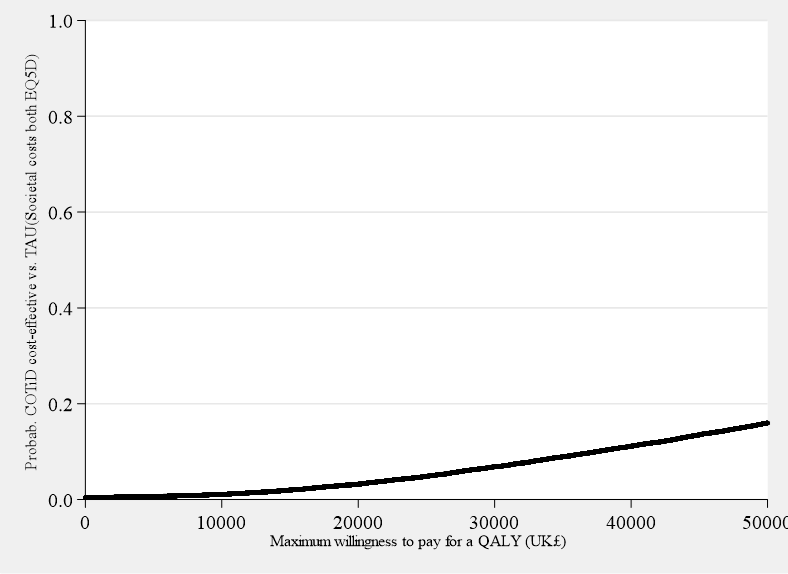


**j) Societal costs both, DEMQOL**


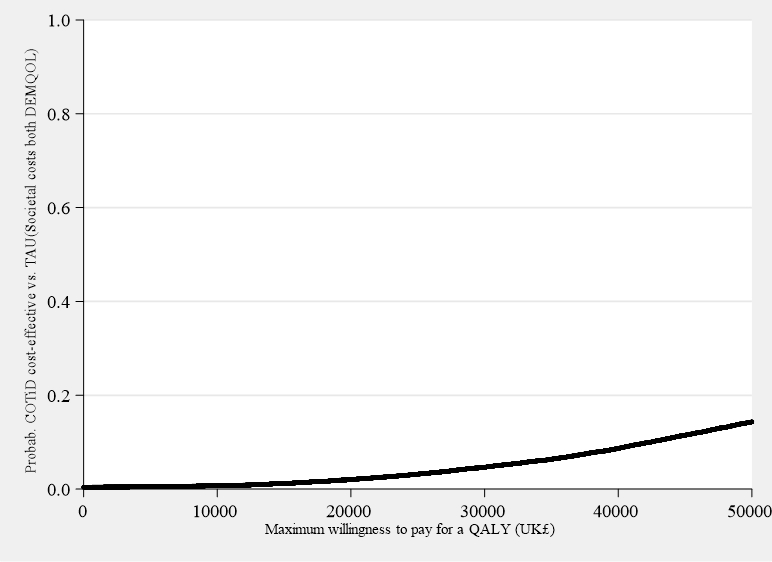

Supplement: S2 Fig — Cost-effectiveness acceptability curve showing the probability that COTiD-UK vs TAU is cost-effective at different values of the maximum willingness to pay for a QALY (a-j). QALY = quality adjusted life year. The probability TUA is cost-effective is one minus the probability COTiD-UK is cost-effective at each value of the maximum willingness to pay for a QALY. (DOCX) [file pone.0262828.s002.docx]
