# Supplementary material for: Cost-utility analysis of community occupational therapy in dementia (COTiD-UK) versus usual care: Results from VALID, a multi-site randomised controlled trial in the UK
Source: PLoS One. 2022 Feb 11;17(2):e0262828. doi: 10.1371/journal.pone.0262828 (PMC8836304; doi:10.1371/journal.pone.0262828)

**S3 Fig. Cost-effectiveness Plane showing the incremental cost (£UK) and the incremental QALYs of COTiD-UK vs TAU.**

QALY=quality adjusted life year. The two dotted lines represent the lower and upper threshold of £20000 and £30000 per QALY. Interventions below the line (threshold) are acceptable, above the line are not acceptable.

1. **NHS costs person with dementia only, DEMQOL (black), EQ-5D-5L (white) and DEMQOL-Proxy (grey)**


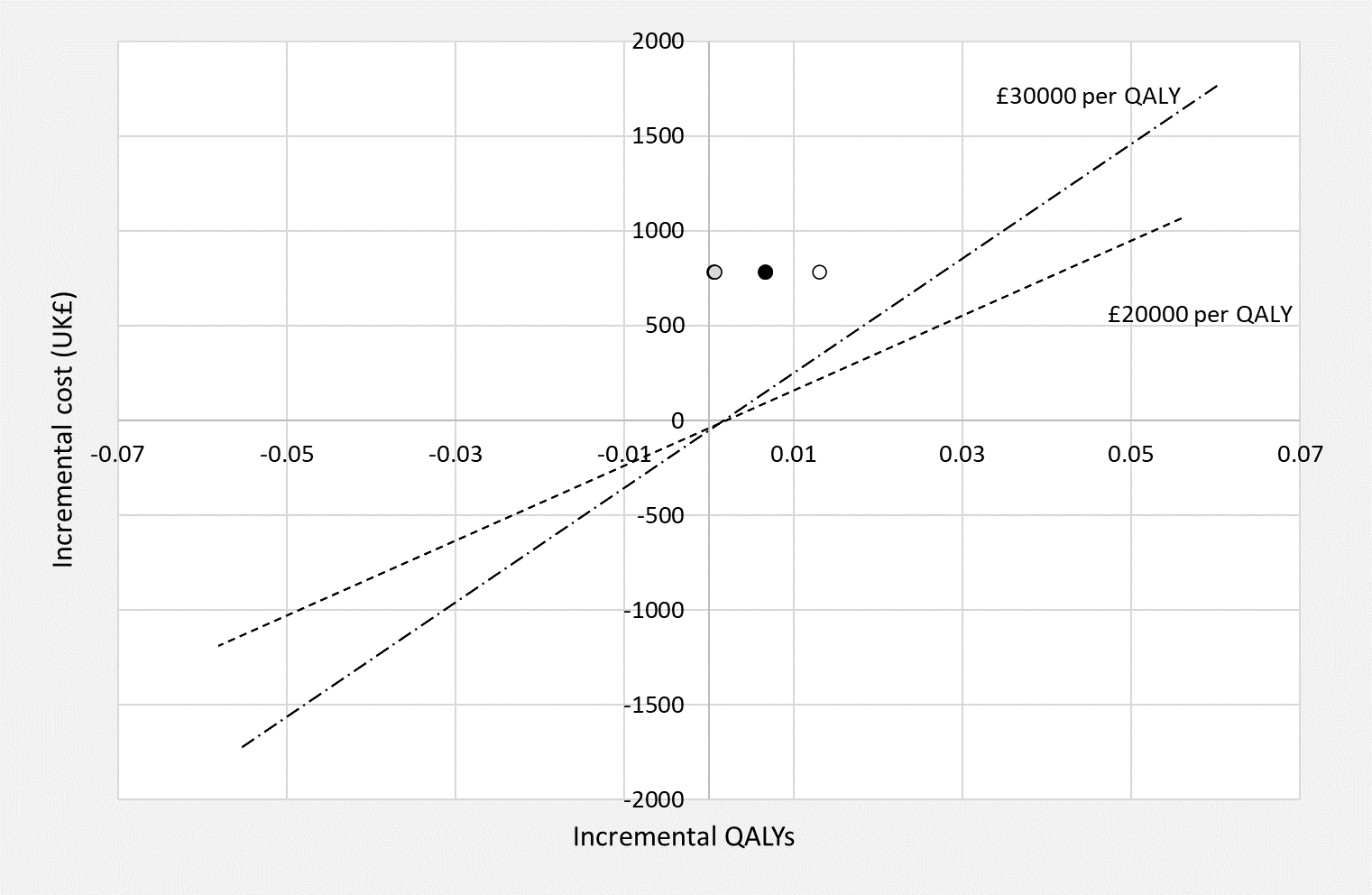


1. **Societal costs person with dementia only, DEMQOL (black), EQ-5D-5L (white) and DEMQOL-Proxy (grey)**


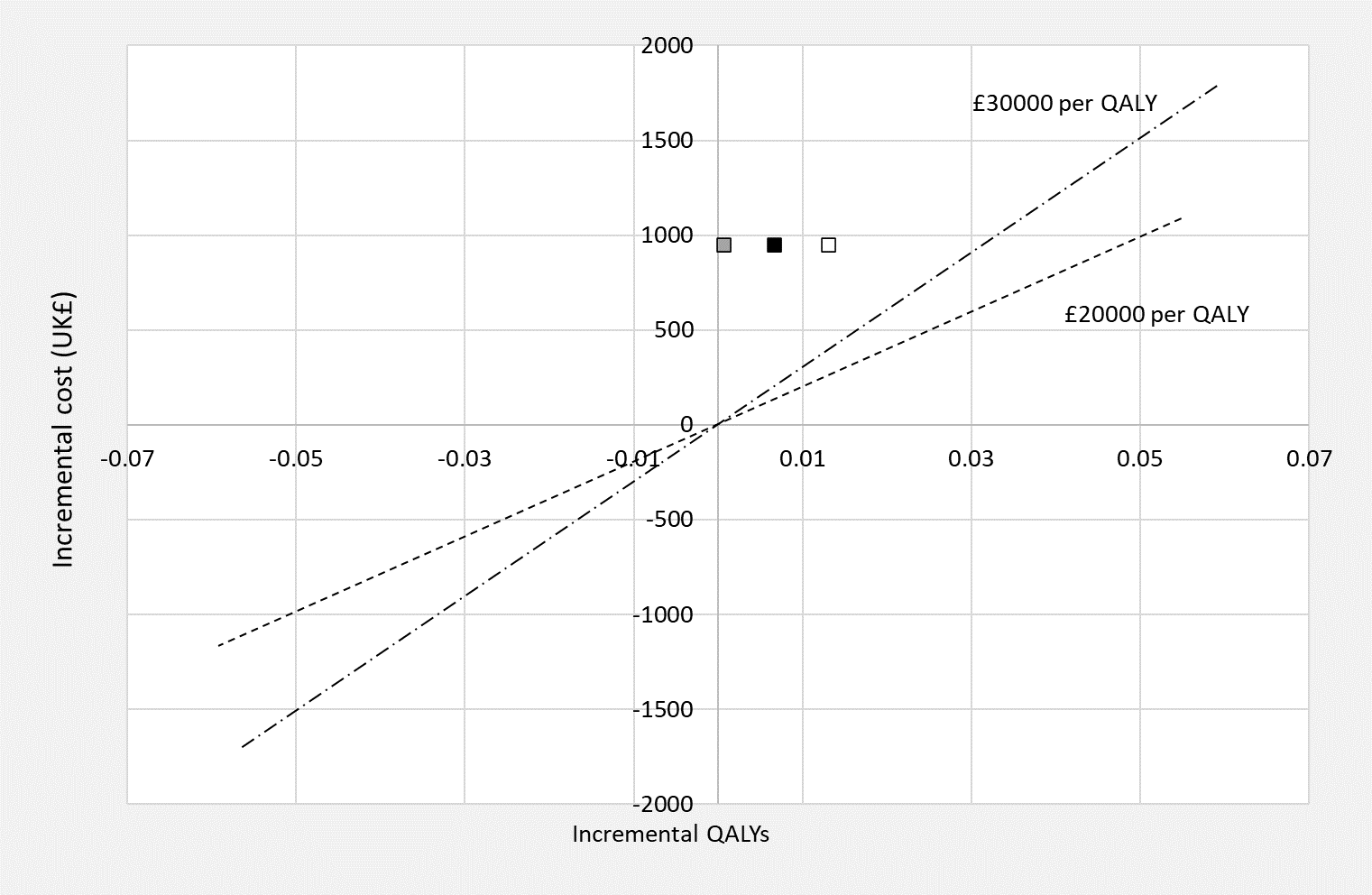


1. **NHS costs both, DEMQOL (black), EQ-5D-5L (white) and DEMQOL-Proxy (grey)**


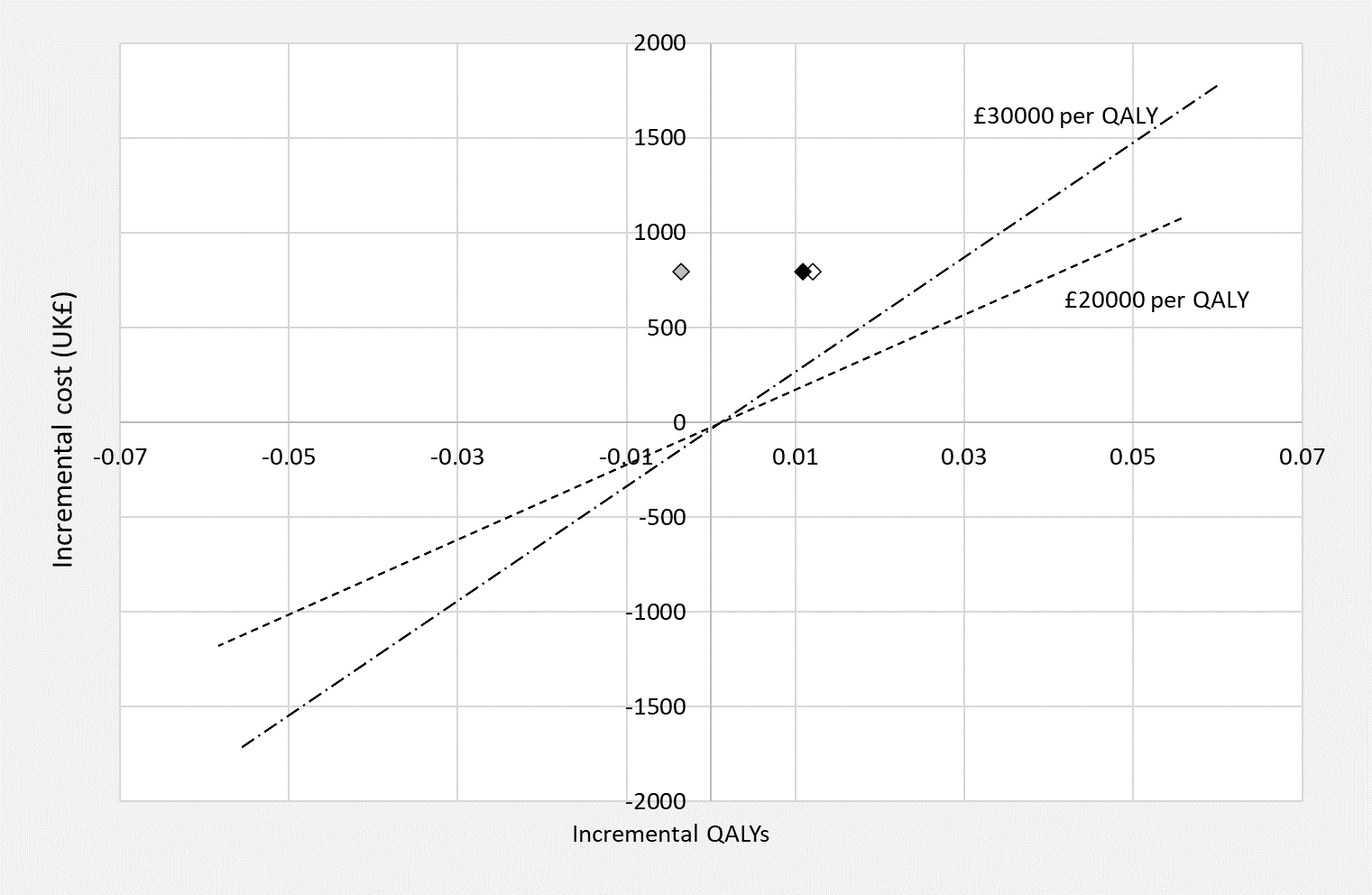


1. **Societal costs both, DEMQOL (black), EQ-5D-5L (white) and DEMQOL-Proxy (grey)**


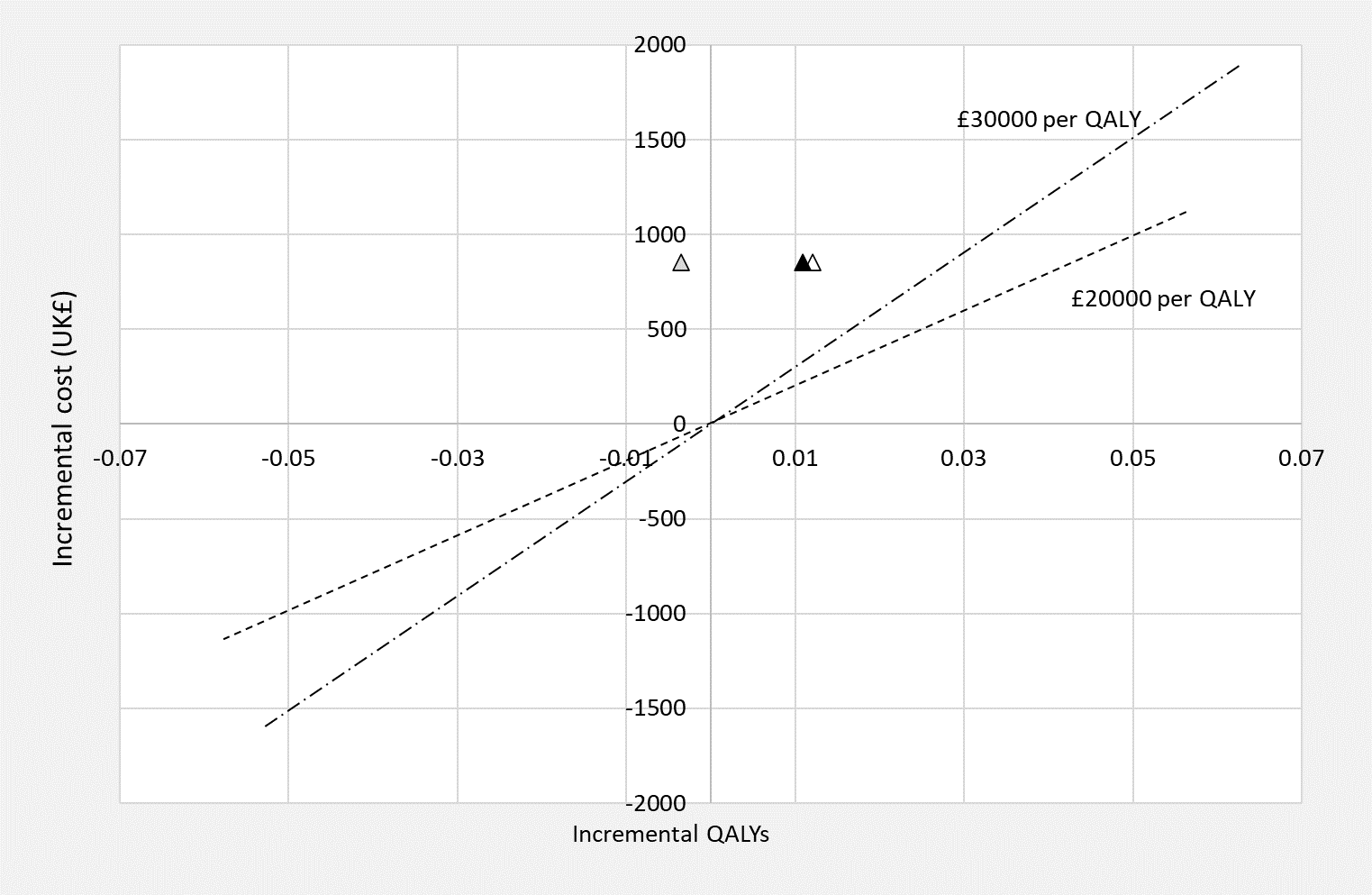

Supplement: S3 Fig — Cost-effectiveness Plane showing the Incremental Costs and Incremental QALYs of COTiD-UK vs TAU (a-d). QALY = quality adjusted life year. The two dotted lines represent the lower and upper threshold of £20000 and £30000 per QALY. Interventions below the line (threshold) are acceptable, above the line are not acceptable. (DOCX) [file pone.0262828.s003.docx]
