# Supplementary material for: Cost-utility analysis of community occupational therapy in dementia (COTiD-UK) versus usual care: Results from VALID, a multi-site randomised controlled trial in the UK
Source: PLoS One. 2022 Feb 11;17(2):e0262828. doi: 10.1371/journal.pone.0262828 (PMC8836304; doi:10.1371/journal.pone.0262828)
Supplement: S1 Appendix — (DOCX) [file pone.0262828.s004.docx]

**S1 *Appendix Cost to train occupational therapists to deliver COTiD-UK***

We assessed the cost of the COTiD-UK set up and training occupational therapists (OTs) in each setting, including venue hiring, refreshment, trainers cost (time, transport and accommodation), occupational therapists costs (time, paid expenses and materials (audio recorder, memory sticks and cost to deliver by post, bag, OPHI, audio feedback and supervision) (S1***Table*** ).
