# Supplementary material for: Cost-utility analysis of community occupational therapy in dementia (COTiD-UK) versus usual care: Results from VALID, a multi-site randomised controlled trial in the UK
Source: PLoS One. 2022 Feb 11;17(2):e0262828. doi: 10.1371/journal.pone.0262828 (PMC8836304; doi:10.1371/journal.pone.0262828)
Supplement: S2 Appendix — (DOCX) [file pone.0262828.s005.docx]

**S2 Appendix Cost of COTiD-UK intervention**

A total of 249 pairs (person with dementia and carer) were allocated to the COTiD-UK intervention arm.

For each pair we collected data on the number and length of COTiD-UK sessions received (including the preparation and recording time), and the time and cost of transport for the occupational therapist (S2***Table*** ).

The cost of COTiD-UK sessions was £131 079 (average £539 per pair) (S2 Table). The cost of transport for occupational therapists was £19 538 (average £80 per pair) (S2 Table).

The total cost of the COTiD-UK intervention (including sessions and transport cost) was £150 617 and was allocated to each person with dementia and included in the cost-utility analysis.
