# Supplementary material for: Cost-utility analysis of community occupational therapy in dementia (COTiD-UK) versus usual care: Results from VALID, a multi-site randomised controlled trial in the UK
Source: PLoS One. 2022 Feb 11;17(2):e0262828. doi: 10.1371/journal.pone.0262828 (PMC8836304; doi:10.1371/journal.pone.0262828)
Supplement: S3 Appendix — (DOCX) [file pone.0262828.s006.docx]

**S3 Appendix Summary of data used in the cost-utility analysis**

Resource use data for person with dementia and carers were collected retrospectively using an adapted version of the CSRI at baseline (covering the previous 12 weeks), 12 weeks and 26 weeks. Unit costs were taken from published sources [19] [20] [21] [22] [23]. Costs were calculated in 2017 Pounds sterling (GBP), inflated where necessary ( ***S3*** Table). For each person with dementia and their carer, the cost of health care resource use was assessed multiplying the number of contacts by the unit cost of each contact. If the average length of contact (in minutes) was reported, the cost was assessed multiplying the average length of contact by the unit cost per minute by the number of contacts (e.g. 20 contacts with a GP, average 10 minutes = 20 x (£242 per hour/60 min x 10 min = £806).

The cost of changes in accommodation were assessed using the number of days spent by each person with dementia in private nursing home (£119 per day), private sector residential care (£94 per day), Local Authority residential care (£162 per day), sheltered housing or supporting lodging (£26.08 per day) as reported in the CSRI at each time point. The following changes in accommodation were not included: days spent in owned or privately rented flat/house, days spent in hotel as part of holidays, days spent at relatives’ place, days spent in hospital wards (already included in NHS resource use).

Medications were reported at each time point for both the person with dementia and carer: 170 different medications/dosages were reported for person with dementia, (mainly used to treat dementia, memory loss, anxiety, depression, sleep, pain, Parkinson’s disease, epilepsy) and 30 different medications/dosages for carers (mainly psychotropic medications used to treat conditions strictly related to care, such as anxiety, depression and mental health problems). For each person the cost of medication was assessed, multiplying the reported dosages (e.g. 2 capsules once a day for one week) by the unit cost of each medication (e.g. cost per one capsule) using the unit cost data in the British National Formulary (BNF) 2017 [19]. When the specific name of the medication was not reported (e.g. “drug for depression”) we assumed it was the most common one used for that specific problem. When dosage was not reported, we assumed it was the one recommended in the BNF. If the dosage reported was “when needed” we assumed the drug was consumed once a week, or as other person with dementia or carers. When the price for a specific medication or dosage was not available in the BNF, we used the price of the lowest/closest dosage or assumed a proportional cost (e.g. “drug x” 15 mg not available, then we used “medication x” 10 mg or half the price of a “medication x” 30 mg). If a person was consuming a medication as part of another trial, but it was not known if it was placebo or treatment, we did not cost it. For participants over 60 years, if not stated otherwise, we assumed medications were NHS costs (i.e., not paid for out of the participants own pocket over the counter).

The cost of adaptations (e.g. a fixed alteration to service users’ home as a consequence of dementia, such as toilet raiser, bath/bed/stair handrail, stair lift, walk-in shower etc.), equipment (e.g. hospital bed, mattress, wheelchair etc.) and continence products/equipment (e.g. incontinence pads, pants, urine bottles, waterproof sheets etc.) were assessed using the resource use data reported in the CSRI at each time point and calculating the cost of each item using the most accurate unit cost source obtained from market sources. When adaptations, equipment and continence products were provided by NHS, LA, council or OTs we assumed they were NHS & PSS costs, when paid privately we included them in the private/societal costs.

Productivity losses for both people with dementia and carers were assessed by multiplying the days off work reported in the CSRI by the average cost of one day of work (day salary) as per ONS 2017 [21].

Transport cost for carers to reach the person with dementia were assessed using the resource use data collected in the trial using the CSRI about the transport used (public transport, car, walking) and the reported cost (per public transport) or the miles per unit cost per mile (car).
