# Supplementary material for: Cost-utility analysis of community occupational therapy in dementia (COTiD-UK) versus usual care: Results from VALID, a multi-site randomised controlled trial in the UK
Source: PLoS One. 2022 Feb 11;17(2):e0262828. doi: 10.1371/journal.pone.0262828 (PMC8836304; doi:10.1371/journal.pone.0262828)
Supplement: S4 Appendix — (DOCX) [file pone.0262828.s007.docx]

**S4 Appendix Results of cost-utility analysis using different perspectives (NHS or societal) and health care questionnaires (EQ-5D-5L, DEMQOL and DEMQOL-Proxy) for person with dementia.**

We performed the cost-utility analysis of COTiD-UK versus TAU assuming different perspectives (NHS/PSS, societal, person with dementia only, person with dementia plus carer):

1. including the NHS cost for person with dementia only (NHS person with dementia only) - (base case analysis);
2. including the NHS and private costs for person with dementia (Societal costs person with dementia only);
3. including the NHS cost for carer (NHS costs for both);
4. including the NHS and private costs for both person with dementia and carer (Societal cost for both).

For each perspective we also combined QALYs for person with dementia assessed using different questionnaires:

1. EQ-5D-5L;
2. DEMQOL (base case analysis);
3. DEMQOL-Proxy.

While we present the base case analysis adopting the NHS perspective for person with dementia only, using the DEMQOL to assess QALYs, we also performed other 11 analyses as a result of the above scenarios. The results of those 11 analyses are presented in the following tables (S4-14 Table**).**

For each analyses we present the cost-effectiveness acceptability curve of COTiD-UK versus TAU for different values of the willingness to pay (***S2*** Figure).
