# Supplementary material for: Cost-utility analysis of community occupational therapy in dementia (COTiD-UK) versus usual care: Results from VALID, a multi-site randomised controlled trial in the UK
Source: PLoS One. 2022 Feb 11;17(2):e0262828. doi: 10.1371/journal.pone.0262828 (PMC8836304; doi:10.1371/journal.pone.0262828)
Supplement: S1 Table — Costs are in 2017 Pounds Sterling (GBP); OT: occupational therapist. (DOCX) [file pone.0262828.s008.docx]

**S1 Table Cost to set-up and train occupational therapists for COTiD-UK**

| Days/events | **1 day Refresh.** | | **1 day Refresh.** | | **2 days Training** | **2 days Training** | **2 days Training** | **2 days Training** | **2 days Training** | **2 days Training** | **1 day Training** | **1 day Training** | **1 day Training** | **2 days Training** | **1 day Training** |
| --- | --- | --- | --- | --- | --- | --- | --- | --- | --- | --- | --- | --- | --- | --- | --- |
| Nr of Trainers (salary band 8 and 7) | 1 Uni (8)  1 Uni Prof | | 1 NHS (7)  1 Uni (7) | | 1 NHS (7) 1 Uni (8) | 1 NHS (7) | 1 NHS (7) | 1 NHS (7) 1 Uni (8) | 1 NHS (7) | 1 NHS (7) | 1 NHS (7) | 1 NHS (7) | 1 NHS (7) 1 Uni (7) | 1 NHS (7) | 1 NHS (7) 1 Uni (8) |
| Nr OTs (salary band 5-6-7-8 a and b) | 1 (5) 2 (6) 1 (7) | | 1 (8a) 1 (7) 1 6) | | 3 6) | 2(7) | 3 (6) | 9 6) 2 (7) 1 (8b) | 3 (6) | 2 (5) 2 (6) 1 (7) | 2 (6) | 7 (6)  1 (7) | 1 (6) 1 (7) 1 (8a) | 5 (6) 1 (7) | 1 (5)  5 (6) 2(7) |
| OT hours per day (days) | 6h (1) | | 6h (1) | | 6h (2) | 6h (2) | 6h (2) | 6h (2) | 6h (2) | 6h (2) | 6h (1) | 6h (1) | 6h (1) | 6h (2) | 6h (1) |
| **OTs time cost** | £1,032 | | £1,074 | | £1,548 | £1,272 | £1,548 | £6,804 | £1,548 | £2,568 | £516 | £2,124 | £948 | £3,216 | £2,124 |
| No. of supervisors/PIs trained | 1 | | 1 | | 1 | 1 | 1 | 3 | 2 | 0 | 1 | 3 | 1 | 2 | 1 |
| Expenses reimbursed to OTs |  | |  | |  |  |  |  |  |  |  | £167 |  | £314 | £296 |
| Supervisors cost | £318 | | £318 | | £636 | £636 | £636 | £1,908 | £1,272 |  | £318 | £954 | £318 | £1,272 | £318 |
| **Trainer 1 time & costs:** | 12 | | 12 | | 20 | 20 | 25 | 20 | 28 | 20 | 18 | 10 | 12 | 20 | 20 |
| Time (incl. travel time and prep) hours | £888 | | £744 | | £1,060 | £1,060 | £1,325 | £1,060 | £1,484 | £1,060 | £954 | £530 | £636 | £1,060 | £1,060 |
| Travel | £130 | | £77 | | £14 | £14 | £65 | £14 | £102 | £14 | £91 | £7 | £67 |  | £13 |
| Overnight subsistence | £61 | | £98 | |  |  | £138 |  | £137 |  | £78 |  | £59 |  |  |
| **Trainer 2 time & costs:** | 10 | | 7 | | 20 |  |  | 20 |  |  |  |  | 7 |  | 20 |
| Time (incl. travel) | £870 | | £434 | | £1,480 |  |  | £1,480 |  |  |  |  | £434 |  | £1,480 |
| Travel cost | £33 | |  | | £14 |  |  | £14 |  |  |  |  |  |  | £13 |
| Venue and refreshment | NHS - not charged | | NHS - not charged | | £1,802 | Uni - not charged | £77 | £1,692 | NHS - not charged | NHS - not charged | NHS - not charged | £799 | £67 | £1,223 | £1,652 |
|  |  | |  | |  |  |  |  |  |  |  |  |  |  |  |
| **TOTAL COST PER EVENT** | **£3,332** | | **£ 2,745** | | **£6,554** | **£2,982** | **£3,789** | **£12,972** | **£4,543** | **£3,642** | **£1,957** | **£4,580** | **£2,530** | **£7,085** | **£6,957** |
|  |  | |  | |  |  |  |  |  |  |  |  |  |  |  |
| TOTAL COST OF TRAINING | | **£63,667** | |  |  |  |  |  |  |  |  |  |  |  |  |
| OT variable costs material | | **£32,802** | |  |  |  |  |  |  |  |  |  |  |  |  |
| **TOTAL COTiD-UK SET UP** | | **£96,469** | |  |  |  |  |  |  |  |  |  |  |  |  |

Note: Costs are in 2017 Pounds sterling (GBP); OT: occupational therapist
