# Supplementary material for: Cost-utility analysis of community occupational therapy in dementia (COTiD-UK) versus usual care: Results from VALID, a multi-site randomised controlled trial in the UK
Source: PLoS One. 2022 Feb 11;17(2):e0262828. doi: 10.1371/journal.pone.0262828 (PMC8836304; doi:10.1371/journal.pone.0262828)
Supplement: S2 Table — Costs are in 2017 Pounds Sterling (GBP); OT: occupational therapist. (DOCX) [file pone.0262828.s009.docx]

**S2 Table Cost of COTiD-UK intervention (OT time per session and OT transport cost)**

| **COTiD-UK intervention** | **Total (N=249)** | **Mean per pair** |
| --- | --- | --- |
| Nr of COTiD-UK sessions | 1955 | 8 |
| Time of sessions (intervention only) | 2124 hours | 8.7 hours |
| Total time of OT session  (including preparation and travel) | 4827 hours | 20 hours |
| Cost of COTiD-UK sessions | £ 131 079 | £ 539 |
| Cost of OT transport to deliver the intervention | £ 19 538 | £ 80 |
| *Total Cost of COTiD-UK intervention* | *£ 150 617* | *£ 619* |

Note: Costs are in 2017 Pounds sterling (GBP); OT: occupational therapist
