# Supplementary material for: Cost-utility analysis of community occupational therapy in dementia (COTiD-UK) versus usual care: Results from VALID, a multi-site randomised controlled trial in the UK
Source: PLoS One. 2022 Feb 11;17(2):e0262828. doi: 10.1371/journal.pone.0262828 (PMC8836304; doi:10.1371/journal.pone.0262828)
Supplement: S3 Table — a) average number of contacts/visits per person; b) Duration of each visit/contact in minutes; c) Total number of contacts in the group; Unit costs are in 2017 Pounds sterling (GBP). Part 3. Resource use, unit costs, utility values, QALYs carer (part 3). a) average number of contacts/visits per person; b) Duration of each visit/contact in minutes; c) Total number of contacts in the group; Unit costs are in 2017 Pounds sterling (GBP). (ZIP) [file pone.0262828.s010.zip › S3_Table part 1_2.docx]

**S3 Table Resource use, unit costs, utility values, QALYs person with dementia (part 1)**

|  | **COTiD-UK** | | | | | | | | |  | **TAU** | | | | | | | | | **Unit cost** |
| --- | --- | --- | --- | --- | --- | --- | --- | --- | --- | --- | --- | --- | --- | --- | --- | --- | --- | --- | --- | --- |
|  | **Baseline (N=249)** | | | **12 weeks (N=211)** | | | **26 weeks (N=198)** | | |  | **Baseline(N=219)** | | | **12 weeks (N=181)** | | | **26 weeks (N=155)** | | | **Source** |
|  | **Aver^a^** | **Dur**^b^ | **Total ^c^** | **Aver^a^** | **Dur**^b^ | **Total^c^** | **Aver^a^** | **Dur**^b^ | **Total^c^** |  | **Aver^a^** | **Dur**^b^ | **Total^c^** | **Aver^a^** | **Dur**^b^ | **Total^c^** | **Aver^a^** | **Dur**^b^ | **Total^c^** |  |
|  |  |  |  |  |  |  |  |  |  |  |  |  |  |  |  |  |  |  |  |  |
| Accident & Emergency. visits | 0.14 |  | 35 | 0.18 |  | 39 | 0.21 |  | 43 |  | 0.21 |  | 46 | 0.1 |  | 19 | 0.07 |  | 11 | £ 141.18 |
| Walk-in center visits | 0.032 |  | 8 | 0.014 |  | 3 | 0.035 |  | 7 |  | 0.05 |  | 11 | 0.06 |  | 11 | 0.2 |  | 31 | £ 141.18 |
| Hospital inpatient ward - acute (days) | 0.34 |  | 85 | 0.3 |  | 63 | 0.69 |  | 138 |  | 0.26 |  | 57 | 0.29 |  | 53 | 0.19 |  | 30 | £ 224.50 |
| Community Hospital ward (days) | 0.028 |  | 7 | 0.004 |  | 1 | 0.005 |  | 1 |  | 0.018 |  | 4 | 0.25 |  | 46 | 0 |  | 0 | £ 224.50 |
| Outpatient visit 1 | 0.64 |  | 159 | 0.7 |  | 145 | 0.55 |  | 110 |  | 0.8 |  | 173 | 0.5 |  | 91 | 0.61 |  | 95 | £ 153.96 |
| Outpatient visit 2 | 0.17 |  | 44 | 0.15 |  | 32 | 0.14 |  | 29 |  | 0.24 |  | 53 | 0.1 |  | 19 | 0.14 |  | 22 | £ 153.96 |
| Outpatient visit 3 | 0.084 |  | 21 | 0.04 |  | 10 | 0.08 |  | 17 |  | 0.04 |  | 10 | 0.05 |  | 10 | 0.051 |  | 8 | £ 153.96 |
| Memory clinic | 0.78 |  | 195 | 0.59 |  | 125 | 0.4 |  | 77 |  | 0.88 |  | 194 | 0.48 |  | 87 | 0.35 |  | 55 | £ 435.00 |
| Day hospital | 0.05 |  | 13 | 0.033 |  | 7 | 0.05 |  | 10 |  | 0.07 |  | 15 | 0.022 |  | 4 | 0.025 |  | 4 | £ 224.50 |
| Other hospital services | 0.068 |  | 17 | 0.014 |  | 3 | 0.44 |  | 88 |  | 0.13 |  | 30 | 0.16 |  | 30 | 0.16 |  | 25 | £ 153.96 |
| Daycare LA attendance (number) | 0.45 | 62 | 113 | 0.75 | 70 | 156 | 1 | 77 | 200 |  | 0.39 | 62 | 86 | 0.96 | 61 | 169 | 0.5 | 107 | 77 | £ 14.00 |
| Day care voluntary organisation | 0.83 | 89 | 208 | 0.82 | 59 | 171 | 1.5 | 86 | 304 |  | 1.21 | 79 | 265 | 1 | 53 | 175 | 0.61 | 72 | 94 | £ 14.00 |
| Day care NHS (community-based) | 0.08 | 20 | 20 | 0.062 | 14 | 13 | 0.11 | 5 | 22 |  | 0.22 | 18 | 48 | 0.23 | 26 | 41 | 0.23 | 18 | 36 | £ 14.00 |
| Lunch club | 0.36 | 40 | 91 | 0.67 | 40 | 139 | 0.49 | 48 | 98 |  | 0.36 | 54 | 80 | 0.44 | 50 | 78 | 0.3 | 41 | 46 | £ 5.00 |
| Social club | 1.67 | 96 | 418 | 1.8 | 72 | 388 | 1.9 | 91 | 385 |  | 1.5 | 85 | 328 | 1.2 | 71 | 222 | 2 | 93 | 314 | £ 5.00 |
| Patient education exercise class | 0.36 | 47 | 90 | 0.62 | 35 | 129 | 0.42 | 38 | 84 |  | 0.63 | 57 | 139 | 0.87 | 64 | 153 | 0.72 | 71 | 112 | £ 28.00 |
| Exercise classes | 0.69 | 21 | 174 | 1.3 | 25 | 270 | 1.28 | 26 | 255 |  | 0.41 | 18 | 91 | 0.85 | 22 | 149 | 0.76 | 22 | 117 | £ 28.00 |
| Other services (Alzheimer café) | 0.72 | 53 | 180 | 0.82 | 59 | 167 | 1.34 | 63 | 264 |  | 0.82 | 52 | 181 | 1.3 | 59 | 225 | 1.3 | 69 | 203 | varies |
| Social worker or Care manager | 0.1 | 13 | 25 | 0.11 | 15 | 23 | 1.37 | 13 | 273 |  | 0.2 | 14 | 85 | 0.08 | 14 | 14 | 0.85 | 21 | 131 | £ 82.00 |
| Home care/home help worker | 14 | 45 | 3397 | 8.69 | 50 | 1808 | 15 | 53 | 3063 |  | 16 | 50 | 3540 | 16 | 31 | 2793 | 16 | 45 | 2476 | £ 26.00 |
| Night sitter carer | 0.22 | 45 | 55 | 0.355 | 15 | 74 | 0.42 | 17 | 84 |  | 0 | 0 | 0 | 0.08 | 26 | 15 | 2.35 | 8 | 360 | £ 27.00 |
| Sitting scheme | 0.76 | 21 | 190 | 1.2 | 34 | 257 | 0.34 | 21 | 69 |  | 0.33 | 23 | 73 | 0.49 | 27 | 87 | 0.39 | 35 | 60 | £ 27.00 |
| Meals on wheels | 1.1 | 2 | 294 | 1.7 | 2 | 356 | 1.13 | 2 | 261 |  | 0.21 | 2 | 48 | 0.32 | 2 | 56 | 0.54 | 2 | 84 | £ 6.72 |
| Laundry service | 0 |  | 0 | 0.15 |  | 33 | 0.35 |  | 7 |  | 0.02 |  | 6 | 0.17 |  | 30 | 0.78 |  | 12 | £ 14.00 |
| Psychiatrist. | 0.18 | 20 | 46 | 0.07 | 7 | 15 | 0.04 | 6 | 9 |  | 0.21 | 28 | 48 | 0.08 | 13 | 14 | 0.084 | 16 | 13 | £ 108.00 |
| Community mental health nurse | 0.17 | 31 | 43 | 0.07 | 9 | 16 | 0.025 | 6 | 5 |  | 0.28 | 35 | 61 | 0.19 | 18 | 34 | 0.14 | 19 | 22 | £ 36.00 |
| Psychologist | 0.08 | 13 | 20 | 0.024 | 6 | 5 | 0.035 | 14 | 7 |  | 0.04 | 5 | 9 | 0.04 | 6 | 8 | 0.032 | 27 | 5 | £ 55.00 |
| Note: a) Average number of contacts/visits per person; b) Duration of each visit/contact in minutes; c) Total number of contacts in the group; Unit costs are in 2017 Pounds sterling (GBP). | | | | | | | | | | | | | | | | | | | | |

**S3 Table Resource use, unit costs, utility values, QALYs person with dementia (part 2)**

|  | **COTiD-UK** | | | | | | | | | |  | **TAU** | | | | | | | | | **Unit cost** |
| --- | --- | --- | --- | --- | --- | --- | --- | --- | --- | --- | --- | --- | --- | --- | --- | --- | --- | --- | --- | --- | --- |
|  | **Baseline (N=249)** | | | | **12 weeks (N=211)** | | | **26 weeks (N=198)** | | |  | **Baseline(N=219)** | | | **12 weeks (N=181)** | | | **26 weeks (N=155)** | | | **Source** |
|  | **Aver^a^** | **Dur**^b^ | **Total^c^** | **Aver^a^** | | **Dur**^b^ | **Total^c^** | **Aver^a^** | **Dur**^b^ | **Total^c^** |  | **Aver^a^** | **Dur**^b^ | **Total^c^** | **Aver^a^** | **Dur**^b^ | **Total^c^** | **Aver^a^** | **Dur**^b^ | **Total^c^** |  |
| Occupational therapist | 0.31 | 25 | 78 | 0.23 | | 15 | 49 | 0.08 | 14 | 16 |  | 0.21 | 24 | 47 | 0.42 | 17 | 74 | 0.097 | 20 | 15 | £ 45.00 |
| Admiral nurse | 0.012 | 6 | 3 | 0.004 | | 2 | 1 | 0.015 | 3 | 3 |  | 0.05 | 5 | 11 | 0.011 | 1 | 2 | 0.006 | 3 | 1 | £ 89.00 |
| Social worker | 0.28 | 6 | 7 | 0.029 | | 7 | 6 | 0.04 | 8 | 8 |  | 0.077 | 4 | 17 | 0.03 | 4 | 6 | 0.045 | 4 | 7 | £ 82.00 |
| Mental Health Support | 0.05 | 6 | 13 | 0.004 | | 2 | 1 | 0.1 | 6 | 21 |  | 0.022 | 9 | 5 | 0.03 | 3 | 5 | 0.019 | 2 | 3 | £ 9.00 |
| Dementia advisor | 0.188 | 31 | 47 | 0.28 | | 22 | 59 | 0.2 | 23 | 41 |  | 0.026 | 25 | 58 | 0.18 | 22 | 31 | 0.12 | 18 | 19 | £ 9.00 |
| Other mental health professional | 0.08 | 9 | 21 | 0.2 | | 12 | 43 | 0.045 | 7 | 9 |  | 0.02 | 10 | 6 | 0.38 | 14 | 66 | 0.012 | 3.47 | 2 | £ 9.00 |
| Paramedic (ambulance service) | 0.14 | 16 | 35 | 0.12 | | 16 | 25 | 0.13 | 25 | 27 |  | 0.14 | 20 | 32 | 0.11 | 16 | 20 | 0.13 | 16 | 21 | £ 188.00 |
| Community Matron | 0.02 | 4 | 6 | 0.058 | | 0.43 | 12 | 0 | 0 | 0 |  | 0.004 | 1 | 1 | 0 | 0 | 0 | 0 | 0 | 0 | £ 62.00 |
| Community/District Nurse | 0.244 | 10 | 61 | 0.7 | | 7 | 146 | 0.71 | 8 | 141 |  | 2.6 | 10 | 573 | 1.2 | 6 | 219 | 1.3 | 10 | 203 | £ 53.00 |
| Practice Nurse | 0.55 | 8 | 138 | 0.53 | | 6.67 | 110 | 0.64 | 9 | 128 |  | 0.61 | 8 | 134 | 0.55 | 9.8 | 96 | 0.4 | 10 | 60 | £ 42.00 |
| Night visit from nursing staff | 0 | 0 | 0 | 0 | | 0 | 0 | 0 | 0 | 0 |  | 0 | 0 | 0 | 0 | 0 | 0 | 0.032 | 1.36 | 5 | £ 44.00 |
| Specialist nurse | 0.277 | 7 | 69 | 0.39 | | 5 | 80 | 0.25 | 9 | 51 |  | 0.24 | 10 | 54 | 0.29 | 9 | 52 | 0.12 | 6 | 19 | £ 53.00 |
| GP | 1.19 | 12 | 297 | 0.98 | | 12 | 204 | 0.9 | 13 | 177 |  | 1.12 | 13 | 247 | 1 | 11 | 176 | 1 | 12 | 177 | £ 242.00 |
| Dentist | 0.377 | 19 | 94 | 0.5 | | 19 | 105 | 0.4 | 15 | 80 |  | 0.378 | 19 | 83 | 0.42 | 16 | 75 | 0.42 | 16 | 66 | £ 101.00 |
| Optician | 0.25 | 23 | 61 | 0.34 | | 21 | 71 | 0.3 | 26 | 61 |  | 0.27 | 25 | 61 | 0.24 | 19 | 41 | 0.22 | 21 | 35 | £ 57.00 |
| Physiotherapist | 0.31 | 13 | 77 | 0.199 | | 10 | 41 | 0.3 | 17 | 61 |  | 0.31 | 16 | 69 | 0.22 | 11 | 40 | 0.2 | 13 | 30 | £ 34.00 |
| Other Primary Care Services | 0.18 | 11 | 44 | 0.18 | | 12 | 37 | 0.3 | 10 | 64 |  | 0.14 | 10 | 31 | 0.57 | 12 | 99 | 0.13 | 24 | 20 | £ 54.00 |
| Change in accommodation (days) | 2.42 |  | 603 | 2 | |  | 467 | 2.47 |  | 508 |  | 1.82 |  | 399 | 1 |  | 185 | 2 |  | 315 | £ 26- £162 |
| Medications (average cost) UK£ | 24 |  |  | 42 | |  |  | 49 |  |  |  | 28 |  |  | 44 |  |  | 52 |  |  | Varies |
| Adaptations | varies |  |  | varies | |  |  | varies |  |  |  | varies |  |  | varies |  |  | varies |  |  | Varies |
| Productivity loss pwd (cost) UK£ | 3810 |  |  | 2.4 | |  |  | 2.5 |  |  |  | 0 |  |  | 0 |  |  | 0 |  |  | £ 73/day |
| Utility EQ-5D-5L pwd | 0.759 |  |  | 0.785 | |  |  | 0.785 |  |  |  | 0.748 |  |  | 0.762 |  |  | 0.739 |  |  |  |
| Utility DEMQOL pwd | 0.647 |  |  | 0.634 | |  |  | 0.638 |  |  |  | 0.634 |  |  | 0.631 |  |  | 0.603 |  |  |  |
| Utility DEMQOL-Proxy pwd | 0.691 |  |  | 0.662 | |  |  | 0.659 |  |  |  | 0.683 |  |  | 0.661 |  |  | 0.645 |  |  |  |
| QALYs EQ-5D-5L pwd |  |  |  |  | |  |  | 0.387 |  |  |  |  |  |  |  |  |  | 0.380 |  |  |  |
| QALYs DEMQOL pwd |  |  |  |  | |  |  | 0.319 |  |  |  |  |  |  |  |  |  | 0.309 |  |  |  |
| QALYs DEMQOL-Proxy pwd |  |  |  |  | |  |  | 0.333 |  |  |  |  |  |  |  |  |  | 0.330 |  |  |  |
|  |  |  |  |  | |  |  |  |  |  |  |  |  |  |  |  |  |  |  |  |  |
| Note: a) Average number of contacts/visits per person; b) Duration of each visit/contact in minutes; c) Total number of contacts in the group; pwd: person with dementia. Unit costs are in 2017 Pounds sterling (GBP). | | | | | | | | | | | | | | | | | | | | | |
