# Supplementary material for: Cost-utility analysis of community occupational therapy in dementia (COTiD-UK) versus usual care: Results from VALID, a multi-site randomised controlled trial in the UK
Source: PLoS One. 2022 Feb 11;17(2):e0262828. doi: 10.1371/journal.pone.0262828 (PMC8836304; doi:10.1371/journal.pone.0262828)
Supplement: S3 Table — a) average number of contacts/visits per person; b) Duration of each visit/contact in minutes; c) Total number of contacts in the group; Unit costs are in 2017 Pounds sterling (GBP). Part 3. Resource use, unit costs, utility values, QALYs carer (part 3). a) average number of contacts/visits per person; b) Duration of each visit/contact in minutes; c) Total number of contacts in the group; Unit costs are in 2017 Pounds sterling (GBP). (ZIP) [file pone.0262828.s010.zip › S3_Table part 3.docx]

**S3 Table (3) Resource use, unit costs, utility values, QALYs carer (part 3)**

|  | **COTiD-UK** | | | | | | | | |  | **TAU** | | | | | | | | | **Unit cost** |
| --- | --- | --- | --- | --- | --- | --- | --- | --- | --- | --- | --- | --- | --- | --- | --- | --- | --- | --- | --- | --- |
|  | **Baseline (N=249)** | | | **12 weeks (N=214)** | | | **26 weeks (N=205)** | | |  | **Baseline (N= 219)** | | | **12 weeks (N= 181)** | | | **26 weeks (N=159)** | | | **Source** |
|  | **Aver^a^** | **Dur**^b^ | **Total^c^** | **Aver^a^** | **Dur**^b^ | **Total^c^** | **Aver^a^** | **Dur**^b^ | **Total^c^** |  | **Aver^a^** | **Dur**^b^ | **Total^c^** | **Aver^a^** | **Dur**^b^ | **Total^c^** | **Aver^a^** | **Dur**^b^ | **Total^c^** |  |
|  |  |  |  |  |  |  |  |  |  |  |  |  |  |  |  |  |  |  |  |  |
| Accident & Emergency. visits | 0.001 |  | 2 |  |  |  | 0.004 |  | 1 |  | 0.004 |  | 1 |  |  | 0 |  |  |  | £ 141.18 |
| Outpatient visit 1 | 0.06 |  | 17 | 0.07 |  | 15 |  |  |  |  | 0.04 |  | 9 | 0.02 |  | 5 | 0.025 |  | 4 | £ 153.96 |
| Memory clinic |  |  |  |  |  |  |  |  |  |  |  |  |  |  |  |  | 0.013 | 30 | 2 | £ 435.00 |
| Daycare LA attendance (number) | 0.008 | 60 | 2 |  |  |  |  |  |  |  |  |  |  |  |  |  |  |  |  | £ 14.00 |
| Social club | 0.07 | 50 | 18 |  |  |  | 0.13 | 121 | 28 |  | 0.018 | 105 | 4 |  |  |  | 0.31 | 120 | 50 | £ 5.00 |
| Patient education exercise class | 0.001 | 90 | 2 |  |  |  | 0.029 | 127 | 6 |  | 0.018 | 120 | 4 | 0.011 | 60 | 2 | 0.14 | 125 | 23 | £ 28.00 |
| Home care/home help worker | 0.004 | 60 | 1 |  |  |  |  |  |  |  | 0.05 | 4 | 12 |  |  |  | 0.031 | 90 | 5 | £ 26.00 |
| Psychiatrist. | 0.02 | 60 | 4 | 0.14 | 45 | 3 | 0.02 | 60 | 5 |  |  |  |  | 0.016 | 45 | 3 | 0.018 | 60 | 3 | £ 108.00 |
| Community mental health nurse | 0.02 | 60 | 5 |  |  |  |  |  |  |  | 0.05 | 60 | 11 |  |  |  |  |  |  | £ 36.00 |
| Psychologist | 0.02 | 150 | 6 |  |  |  | 0.014 | 30 | 3 |  |  |  |  |  |  |  | 0.006 | 60 | 1 | £ 55.00 |
| Occupational therapist |  |  |  |  |  |  |  |  |  |  |  |  |  |  |  |  | 0.006 | 12 | 1 | £ 45.00 |
| Admiral nurse | 0.02 | 45 | 4 | 0.009 | 60 | 2 | 0.009 | 30 | 2 |  | 0.009 | 52 | 2 | 0.011 | 12 | 2 |  |  |  | £ 89.00 |
| Social worker | 0.004 | 60 | 1 | 0.04 | 110 | 8 | 0.004 | 60 | 1 |  |  |  |  | 0.005 | 15 | 1 | 0.006 | 90 | 1 | £ 82.00 |
| Mental Health Support | 0.23 | 115 | 58 | 0.38 | 120 | 81 | 0.31 | 103 | 64 |  | 0.25 | 109 | 56 | 0.21 | 129 | 39 | 0.11 | 98 | 19 | £ 9.00 |
| Practice Nurse | 0.001 | 10 | 2 | 0.14 | 10 | 3 |  |  |  |  | 0.03 | 14 | 6 | 0.016 | 10 | 3 | 0.025 | 13 | 4 | £ 42.00 |
| Specialist nurse | 0.03 | 60 | 8 |  |  |  |  |  |  |  | 0.004 | 30 | 1 | 0.005 | 10 | 1 |  |  |  | £ 53.00 |
| GP | 0.22 | 14 | 56 | 0.1 | 15 | 22 | 0.11 | 15 | 23 |  | 0.29 | 14 | 50 | 0.09 | 13 | 17 | 0.16 | 14 | 26 | £ 242.00 |
| Dentist |  |  |  | 0.004 | 60 | 1 | 0.004 | 140 | 1 |  |  |  |  |  |  |  |  |  |  | £ 101.00 |
| Optician |  |  |  |  |  |  |  |  |  |  | 0.004 | 30 | 1 |  |  |  |  |  |  | £ 57.00 |
| Physiotherapist | 0.04 | 60 | 9 | 0.04 | 18 | 9 | 0.014 | 45 | 3 |  | 0.07 | 83 | 16 | 0.16 | 26 | 29 | 0.09 | 75 | 15 | £ 34.00 |
| Other Primary Care Services | 0.004 | 12 | 1 |  |  |  | 0.004 | 30 | 1 |  | 0.03 | 26 | 7 | 0.016 | 50 | 3 | 0.06 | 23 | 10 | £ 54.00 |
| Medications (average cost) UK£ | 1.8 |  |  | 2 |  |  | 1.85 |  |  |  | 0.5 |  |  | 0.82 |  |  | 2.6 |  |  | Varies |
| Productivity loss supp (cost) UK£ | 100 |  |  | 23 |  |  | 32 |  |  |  | 81 |  |  | 92 |  |  | 29 |  |  | £ 73/day |
| Transport cost carer UK£ | 7 |  |  | 8 |  |  | 6 |  |  |  | 12 |  |  | 13 |  |  | 9 |  |  | Varies |
| Utility EQ-5D-5L carer | 0.791 |  |  | 0.796 |  |  | 0.784 |  |  |  | 0.789 |  |  | 0.794 |  |  | 0.792 |  |  |  |
| QALYs EQ-5D-5L carer |  |  |  |  |  |  | 0.394 |  |  |  |  |  |  |  |  |  | 0.399 |  |  |  |
|  |  |  |  |  |  |  |  |  |  |  |  |  |  |  |  |  |  |  |  |  |
| Note: a) Average number of contacts/visits per person; b) Duration of each visit/contact in minutes; c) Total number of contacts in the group. Unit costs are in 2017 Pounds sterling (GBP). | | | | | | | | | | | | | | | | | | | | |
