# Supplementary material for: Cost-utility analysis of community occupational therapy in dementia (COTiD-UK) versus usual care: Results from VALID, a multi-site randomised controlled trial in the UK
Source: PLoS One. 2022 Feb 11;17(2):e0262828. doi: 10.1371/journal.pone.0262828 (PMC8836304; doi:10.1371/journal.pone.0262828)
Supplement: S4 Table — a) Data include values imputed using multiple imputation (see text). The QALYs gained, incremental cost and incremental NMB figures are for COTiD-UK minus TAU and are adjusted for potential confounders (see text).; b) As for the base case analysis except the QALYs gained and costs are unadjusted.; c) As for the base case analysis except there is no multiple imputation of missing values.; d) As for c but the analysis is unadjusted. (DOCX) [file pone.0262828.s011.docx]

**S4 Table Incremental cost-effectiveness of COTiD-UK vs. TAU, NHS costs person with dementia using EQ-5D-5L**

|  | Incremental cost | |  | QALYs gained | |  | Incremental Net Monetary Benefit | | | |  |
| --- | --- | --- | --- | --- | --- | --- | --- | --- | --- | --- | --- |
|  |  |  |  |  |  |  | £20,000 |  |  | £30,000 |  |
|  | Mean | 95% CI |  | Mean | 95% CI |  | Mean | 95% CI |  | Mean | 95% CI |
| Base case ^a^ | 784 | ( 233 , 1334) |  | 0.01298 | (-0.00089 , 0.0268) |  | -524 | (-745, -303) |  | -394 | (-642, -147) |
|  |  |  |  |  |  |  |  |  |  |  |  |
| No adjustment ^b^ | 769 | (171, 1368) |  | 0.01716 | (-0.00356, 0.03788) |  | -426 | (-687, -164) |  | -254 | (-564, 56) |
|  |  |  |  |  |  |  |  |  |  |  |  |
| Complete case analysis ^c^ | 838 | (274, 1402) |  | 0.00994 | (-0.004458, 0.02434) |  | -639 | (-867,-412) |  | -540 | (-795, -285) |
|  |  |  |  |  |  |  |  |  |  |  |  |
| Complete case analysis, no adjustment ^d^ | 1047 | (441, 1653) |  | 0.00647 | (-0.01693, 0.02986) |  | -918 | (-1193, -643) |  | -853 | (-1186, -520) |
