# Supplementary material for: Cost-utility analysis of community occupational therapy in dementia (COTiD-UK) versus usual care: Results from VALID, a multi-site randomised controlled trial in the UK
Source: PLoS One. 2022 Feb 11;17(2):e0262828. doi: 10.1371/journal.pone.0262828 (PMC8836304; doi:10.1371/journal.pone.0262828)
Supplement: S5 Table — a) Data include values imputed using multiple imputation (see text). The QALYs gained, incremental cost and incremental NMB figures are for COTiD-UK minus TAU and are adjusted for potential confounders (see text).; b) As for the base case analysis except the QALYs gained and costs are unadjusted.; c) As for the base case analysis except there is no multiple imputation of missing values.; d) As for c but the analysis is unadjusted. (DOCX) [file pone.0262828.s012.docx]

**S5 Table Incremental cost-effectiveness of COTiD-UK vs. TAU, NHS costs person with dementia using DEMQOL-Proxy**

|  | Incremental cost | |  | QALYs gained | |  | Incremental Net Monetary Benefit | | | |  |
| --- | --- | --- | --- | --- | --- | --- | --- | --- | --- | --- | --- |
|  |  |  |  |  |  |  | £20,000 |  |  | £30,000 |  |
|  | Mean | 95% CI |  | Mean | 95% CI |  | Mean | 95% CI |  | Mean | 95% CI |
| Base case ^a^ | 784 | ( 233 , 1334) |  | 0.00063 | (-0.00994 , 0.0112) |  | -771 | (-996, -547) |  | -765 | (-1002, -528) |
|  |  |  |  |  |  |  |  |  |  |  |  |
| No adjustment ^b^ | 769 | (171, 1368) |  | 0.00287 | (-0.00992, 0.01567) |  | -712 | (-959, -464) |  | -683 | (-947,-419) |
|  |  |  |  |  |  |  |  |  |  |  |  |
| Complete case analysis ^c^ | 838 | (274, 1402) |  | 0.00063 | (-0.00994, 0.01119) |  | -825 | (-1042, -609) |  | -819 | (-1052, -587) |
|  |  |  |  |  |  |  |  |  |  |  |  |
| Complete case analysis, no adjustment ^d^ | 1047 | (441, 1653) |  | 0.00288 | (-0.009924, 0.01567) |  | -989 | (-1226, -753) |  | -961 | (-1219, -703) |

1. Data include values imputed using multiple imputation (see text). The QALYs gained, incremental cost and incremental NMB figures are for COTiD-UK minus TAU and are adjusted for potential confounders (see text).
2. As for the base case analysis except the QALYs gained and costs are unadjusted.
3. As for the base case analysis except there is no multiple imputation of missing values.
4. As for c but the analysis is unadjusted.
