# Supplementary material for: Cost-utility analysis of community occupational therapy in dementia (COTiD-UK) versus usual care: Results from VALID, a multi-site randomised controlled trial in the UK
Source: PLoS One. 2022 Feb 11;17(2):e0262828. doi: 10.1371/journal.pone.0262828 (PMC8836304; doi:10.1371/journal.pone.0262828)
Supplement: S15 Table — (DOCX) [file pone.0262828.s022.docx]

**S15 Table Consolidated Health Economic Evaluation Reporting Standards (CHEERS) statement**

| **Section** | **Item no.** | **Reported on** | **Page number(s)** |
| --- | --- | --- | --- |
| **Title and abstract** | | |  |
| Title | 1 | The title identifies the study as an economic analysis and describes the interventions being evaluated. | 1 |
| Abstract | 2 | A structured summary is provided. | 2 |
| **Introduction** | | |  |
| Background and objectives | 3 | The broader context for the study, and the research question and its rationale, are described in the “Introduction”. | 3 |
| **Methods** | | |  |
| Target population and subgroups | 4 | The target population is described in the “VALID Trial Background”. | 4 |
| Setting and location | 5 | The setting and location of the trial are described in the “Trial background” sub-section. | 4 |
| Study perspective | 6 | The study perspective is described in the “Overview of economic evaluation” sub-section. | 6 |
| Comparators | 7 | The comparators are described and justified in the “Introduction”, “Methods”. | 3,4,5 |
| Time horizon | 8 | The time horizon is described and justified in the abstract and in the “Overview of economic evaluation” sub-section. | 6 |
| Discount rate | 9 | Costs and outcomes have not been discounted because the time horizon was less than one year. This is described and justified in the “Overview of economic evaluation” sub-section. | 6 |
| Choice of health outcomes | 10 | The outcome measure is described and justified in the “Trial background” and “Overview of economic evaluation” sub-sections. | 4,5 |
| Measurement of effectiveness | 11a | The clinical trial used to measure effectiveness is described in the “Trial background” sub-section and Utilities and QALYs section | 4, 7 |
| Measurement and valuation of preference based outcomes | 12 | Methods used to measure and value preference based outcomes are described in the “Utilities and QALYs” sub-section. | 7,8 |
| Estimating resources and costs | 13a | Methods used to estimate resources and costs are described in the “Resource use and costs” sub-section and in Supplementary material. | 5,6,7 |
| Currency, price date, and conversion | 14 | Currency, price date and conversion are described in the “Overview of economic evaluation” sub-section. | 7 |
| Choice of model | 15 | We explain that extrapolation beyond the end of the trial using decision-analytical modelling was not undertaken in the “Overview of economic evaluation” sub-section. | 5 |
| Assumptions | 16 | All assumptions used in the analysis are described throughout the “Methods” and the online-only Data Supplement. | 4-8 |
| Analytical methods | 17 | Analytical methods are described in the ‘”Dealing with missing data” and “Statistical methods used to compare costs and outcomes” sub-sections. | 7,8 |
| **Results** | | |  |
| Study parameters | 18 | The main study parameters are in supplementary data S3 Table |  |
| Incremental costs and outcomes | 19 | Incremental costs and outcomes are reported in Tables 1, 2 and Figure 1 and discussed throughout the “Results”. | 10 |
| Characterising uncertainty | 20a | Methods used in the sensitivity analyses are described in the “Sensitivity analyses” sub-section in the “Methods”. The results are presented in Table 1-2 and Figure 1 and discussed in the “Sensitivity analyses” sub-section in the “Results”. | 9, 10 |
| Characterising heterogeneity | 21 | Methods used are described in the “Sensitivity analyses” sub-section in the “Methods”. The results are presented in Table 1-2 and discussed in the “Sensitivity analyses” sub-section in the “Results”. | 9,10 |
| **Discussion** | | |  |
| Study findings, limitations, generalisability, and current knowledge | 22 | Study findings, limitations, generalisability and comparisons with current knowledge are discussed in the “Discussion”. | 14 |
| **Other** | | |  |
| Source of funding | 23 | The funding source and role of the funder is described in the “Financial Disclosure Statement” sub-section. | 15,16 |
| Conflicts of interest | 24 | Conflicts of interests are described in the “Disclosure” sub-section. |  |
